# A Quantitative HILIC–MS/MS Assay of the Metabolic Response of Huh-7 Cells Exposed to 2,3,7,8-Tetrachlorodibenzo-*p*-Dioxin

**DOI:** 10.3390/metabo9060118

**Published:** 2019-06-20

**Authors:** Qing Liu, Jingwei Cai, Robert G. Nichols, Yuan Tian, Jintao Zhang, Philip B. Smith, Yan Wang, Chao Yan, Andrew D. Patterson

**Affiliations:** 1 School of Pharmacy, Shanghai Jiao Tong University, Shanghai 200240, China; qul76@psu.edu (Q.L.); wangyan11@sjtu.edu.cn (Y.W.); chaoyan@sjtu.edu.cn (C.Y.); 2 Center for Molecular Toxicology and Carcinogenesis, Department of Veterinary and Biomedical Sciences, Pennsylvania State University, University Park, PA 16802, USA; vivianna620@gmail.com (J.C.); rgn5011@psu.edu (R.G.N.); yzt11@psu.edu (Y.T.); jingtao.zhang@ntu.edu.sg (J.Z.); 3 CAS Key Laboratory of Magnetic Resonance in Biological Systems, National Centre for Magnetic Resonance in Wuhan, Wuhan Institute of Physics and Mathematics, Chinese Academy of Sciences (CAS), Wuhan 430071, China; 4 Huck Institute of the Life Sciences, University Park, PA 16802, USA; pbs13@psu.edu

**Keywords:** polar metabolites, HILIC–UHPLC–MS/MS, targeted metabolomics, TCDD

## Abstract

A hydrophilic interaction liquid chromatography (HILIC)–ultra high-pressure liquid chromatography (UHPLC) coupled with tandem mass spectrometry (MS/MS) method was developed and applied to profile metabolite changes in human Huh-7 cells exposed to the potent aryl hydrocarbon receptor (AHR) ligand 2,3,7,8-tetrachlorodibenzo-*p*-dioxin (TCDD). Comparisons of sensitivity (limit of detection as low as 0.01 µM) and reproducibility (84% of compounds had an interday relative standard deviation (RSD) less than 10.0%; 83% of compounds had an intraday RSD less than 15.0%) were assessed for all the metabolites. The exposure of Huh-7 cells to the hepatotoxic carcinogen TCDD at low doses (1 nM and 10 nM for 4 h and 24 h, respectively) was reflected by the disturbance of amino acid metabolism, energy metabolism (glycolysis, TCA cycle), and nucleic acid metabolism. TCDD caused a significant decrease in amino acids such as serine, alanine, and proline while promoting an increase in arginine levels with 24 h treatment. Energy metabolism intermediates such as phosphoenolpyruvate and acetyl–CoA and nucleosides such as UMP, XMP, and CMP were also markedly decreased. These results support the application of HILIC–UHPLC–MS/MS for robust and reliable analysis of the cellular response to environmentally relevant toxicants at lower doses.

## 1. Introduction

Metabolomics aims to understand the metabolic dynamics in living systems and provide a readout of physiologic status [1,2]. Metabolomics has impacted the fields of analytical and clinical chemistry, nutrition, drug discovery, and toxicology [3,4,5,6]. Metabolomics seeks to accurately identify and quantify as many compounds as possible utilizing reference standards to achieve the highest level of annotation confidence.

In toxicology, metabolomics has been an important tool for providing a better view of toxicity mechanisms. Information obtained from metabolomic studies reflecting metabolic changes may be more relevant to classical toxicological endpoints rather than transcriptomics, as metabolic changes are downstream of those initial changes that occur at the level of the genome, transcriptome, and proteome [7].

2,3,7,8-tetrachlorodibenzo-*p*-dioxin (TCDD) is a potent agonist of the aryl hydrocarbon receptor (AHR, a cytosolic ligand-activated transcription factor). TCDD has been classified as a probable human carcinogen by the United States (U.S.) Environmental Protection Agency (EPA), since it may be implicated in human soft-tissue sarcomas, lymphomas, and stomach carcinomas [8,9]. The biological effects of TCDD include adaptive responses, wasting syndrome, tumor promotion, immunotoxicity, developmental toxicity, or endocrine toxicity [10,11,12,13,14]. In most cases, biofluids or tissues from in vivo experiments have been analyzed via metabolomics [15,16], but the development of robust and high-throughput profiling methods of intracellular metabolites using cell culture systems [17,18] is lacking.

Metabolite quantitation and identification remain highly challenging tasks due to the different physicochemical properties of metabolites, their broad range of concentration (fM to mM), and the limited number of trials to systematically compare different methodologies. Herein, we report a hydrophilic interaction liquid chromatography (HILIC)–ultra high-pressure liquid chromatography (UHPLC)–MS/MS approach permitting the fast, selective, and quantitative measurement of polar metabolites involved in critical metabolic pathways influenced by TCDD exposure. In our study, 107 polar metabolites in a wide range of classes were accurately quantified including amino acids (27), organic acids (14), sugars (2), phosphorylated sugars (7), nucleobases (12), nucleotides (19), phosphorylated metabolites (11), hydrophilic vitamins (8), and coenzymes (7). The HILIC method was optimized based on the LC gradient, column temperature, ion concentration, and solvent pH. The quantitation accuracy was evaluated using isotope standards. 

TCDD induces the expression of the AHR target gene cytochrome P450 1A1 (*CYP1A1*) in Huh-7 cells [19]. To characterize the toxicity response to TCDD, the HILIC–UHPLC–MS/MS method was used to examine extracts from Huh-7 cells. Partial least squares discriminant analysis (PLS-DA) identified differences based on TCDD exposure time and doses. In addition, ^1^H NMR-based metabolomics was applied to generate a complementary metabolic perspective in order to validate the UHPLC–HILIC–MS/MS approach. Our findings provide insights into the metabolic impact of TCDD toxicity.

## 2. Results and Discussions

In this study, 107 polar metabolites including components of central carbon, amino acid, and nucleotide metabolism were quantitated by the developed HILIC–MS/MS using stable isotopes as internal standards. In addition to the conventional method validation, the HILIC–MS/MS method was validated with Huh-7 cells for the glycolytic pathway and then applied on TCDD toxicology with Huh-7 Cells.

### 2.1. MRM Transitions and Validation of the HILIC-UHPLC-MS/MS Method

MRM transitions (MT), retention time (RT), linear range (LR), limit of detection (LOD), and limit of quantitation (LOQ) of the 107 compounds from different classes of metabolites are listed in Table S1. A total of 88% of the compounds had a LOD and LOQ value ranging from 0.01 to 1 µM and 0.03 to 3 µM, respectively, on the Waters Xevo TQD mass spectrometer (Milford, MA, USA). Phosphorylated compounds such as E4P (LOD = 7.0 µM, LOQ = 21.0 µM), F1,6BP (LOD = 1.0 µM, LOQ = 3.0 µM), R5P (LOD = 2.0 µM, LOQ = 6.0 µM), Ru5P (LOD = 2.0 µM, LOQ = 6.0 µM), multiple organic acid or citric acid/isocitric acid (LOD = 1.6 µM, LOQ = 4.7 µM) and shikimic acid (LOD = 3.0 µM, LOQ = 9.0 µM) showed relatively higher LOD/LOQ values owing to their low ionization efficiency in MS. Linear calibration curves with R^2^ ranging from 0.99 to 0.999 were obtained for each polar metabolite. The broad linear concentration range ensures the accurate quantitation of polar metabolites in cells and tissues. The intraday injection RSD (N = 5) of the spike-in cell extraction sample ranged from 2.1% to 17.2%, with 84% of RSD below 10%, and the interday precision (N = 5) range from 3.3% to 30.60%, with 83% of RSD below 15%. All the transitions of isotope internal standards and transitions of the compounds are listed in Table S2. Negative ion mode was used for less than one-quarter (23%) of the compounds, and for the remainder (77%) positive ion mode was used. This HILIC–UHPLC–MS/MS method is relatively easy to transfer to other platforms, including the Waters Xevo TQS, which has increased sensitivity.

### 2.2. HILIC Method Development

Biological samples contain isomeric and isobaric polar metabolites such as isoleucine/leucine, glutamine/lysine, F6P/G6P, and these metabolites cannot be differentiated by MS/MS. Therefore, chromatographic separation is essential for accurate quantification [20]. Moreover, maximum chromatographic separation of all the metabolites present in an extract can also prevent or minimize ion suppression. The retention mechanism of HILIC mainly relies on the partition–phase transfer of analytes between an aqueous stationary solvent and relatively non-polar organic mobile phase [21,22,23]. HILIC also involves polar interactions (hydrogen bonding) and ion exchange between analytes and the column stationary phase. In the current study, LC conditions were optimized based on Malik et al. [24] (Table S3). Mobile phase ionic strength, temperature, and stationary phase conditions are key parameters for successful HILIC [25]. Here, the influence of mobile phase gradient, ionic strength, and column temperature were evaluated (Figure S1). Twelve metabolites from different classes were selected, including niacinamide, hippuric acid, creatinine, valine, proline, Vit B_12_, FAD, malic acid, acetyl CoA, AMP, R5P, and GSSG. Hippuric acid and malic acid were acquired in ESI- mode, and the rest were acquired in ESI+ mode. The separation of five pairs of isomers is shown in Figure S2; the five pairs of isomers achieve basic separation (R ≥ 1) in our study, separately. 

### 2.3. Validation of Method with Huh-7 Cells for Glycolysis Pathway

After establishing and optimizing the HILIC method, we aimed to quantify polar metabolites in Huh-7 cells treated with bromopyruvate, which is a glycolysis inhibitor. 3-BrPy [26,27,28], a halogenated form of pyruvate, has high anti-tumor activity as a glycolytic inhibitor, both in vitro and in vivo [29]. The primary target of 3-BrPy in glycolysis appears to be glyceraldehyde-3-phosphate dehydrogenase (GAPDH). Normally, hexokinase isoform II (HK II) [30] is not expressed in most tissues but is highly expressed in many tumor cells including hepatocellular carcinoma (such as Huh-7 cells, HepG2 cells).

Unlike other chemotherapeutic agents, 3-BrPy is less toxic to cells [26]. Ganapathy-Kanniappan et al. [31] found that 3-BrPy suppressed the proliferation of Hep3B cells at 150 µM. Meng et al. [32] showed that the short-term (24–48 h) depletion of either glucose or glutamine resulted in growth arrest, but not the death of Hep3B cells. To avoid excessive cell death, Huh-7 cells were treated with 3-BrPy at concentrations of 5 µM, 15 µM, 50 µM, and 100 µM, and media with low glucose (2 mM) was used as the positive control (all the media was supplemented with 1% sodium pyruvate). Pharmacologic targeting of aerobic glycolysis in Huh-7 cells was shown in Figure S3. According to the MTT results of each sample (Figure S4A), the exposure of Huh-7 cells for 24 h to 5–100 µM of 3-BrPy did not cause obvious cell death, which is similar to that reported from primary rat astrocytes [33]. 

Eighty metabolites were detected and quantified using our method (Table S5). Principal components analysis (PCA) of polar metabolites of Huh-7 cells treated with different concentrations of 3-BrPy is shown in Figure S4B. Heatmap of polar metabolites involved in glycolysis metabolism in the presence of 3-BrPy is shown in Figure S4C. Specifically, the quantitation of important metabolites involved in glycolysis was also analyzed among groups using one-way ANOVA (Figure S5). As expected, intracellular ATP and NADH significantly decreased in a dose-dependent manner after adding 3-BrPy. The important intermediates F6P, F1,6-BP, and 3-phosphoglyceric acid in the glycolysis pathway decreased after treatment with 50 µM and 100 µM of 3-BrPy, phosphoenolpyruvate decreased after treatment with 100 µM of 3-BrPy. These data clearly indicated that the glycolysis pathway in Huh-7 cells was inhibited by 3-BrPy. Unchanged levels of phosphoenolpyruvate in the positive control may be due to feedback in the low glucose environment. Reduced activity of the dimeric form of PKM2 (pyruvate kinase) provides the metabolic advantage to accumulate phosphoenolpyruvate, which provides precursors for the synthesis of amino acids, nucleic acids, and lipids, thus providing cancer cells with a growth advantage [34]. The intermediate products in the TCA cycle showed no significant change in 3-BrPy treatment, indicating little influence on TCA by 3-BrPy with a concentration below 100 µM. Glutamine and glucose represent two important nutrients for proliferating cells; the decrease of glutamine indicated the weakened activity in uptake due to glycolysis blockage [35]. Glucogenic amino acid synthesis including alanine decreased, while ketogenic amino acid leucine increased in this experiment. Reduced glutathione (GSH) and glutathione oxidized (GSSG) had no significant changes in our research, suggesting that unlike astrocytes [33], 3-BrPy may not deplete the GSH of Huh-7 cells as rapidly. As expected, glycolysis in Huh-7 cells was inhibited by 3-BrPy, indicating that the UHPLC–HILIC–MS/MS method was reliable. Then, this method was applied to understand low-dose TCDD toxicity.

### 2.4. Application of Method on TCDD Toxicology with Huh-7 Cells

TCDD has been widely studied in vivo and in vitro, particularly in terms of its hepatotoxic, carcinogenic, and physiological effects [36,37], which are mediated by the AHR. Transcriptomic and proteomic approaches for TCDD toxicity testing have been widely applied in vitro [8,19] (usually approximately 10 nM to 10,000 nM), but relatively few studies have used metabolomics, particularly at low doses that are unlikely to elicit similar responses as doses not typically found with environmental exposures. Hence, in our study, Huh-7 cells were treated with TCDD at two lower concentrations of 1 nM and 10 nM for 4 h and 24 h, respectively. 

**Figure 1:**
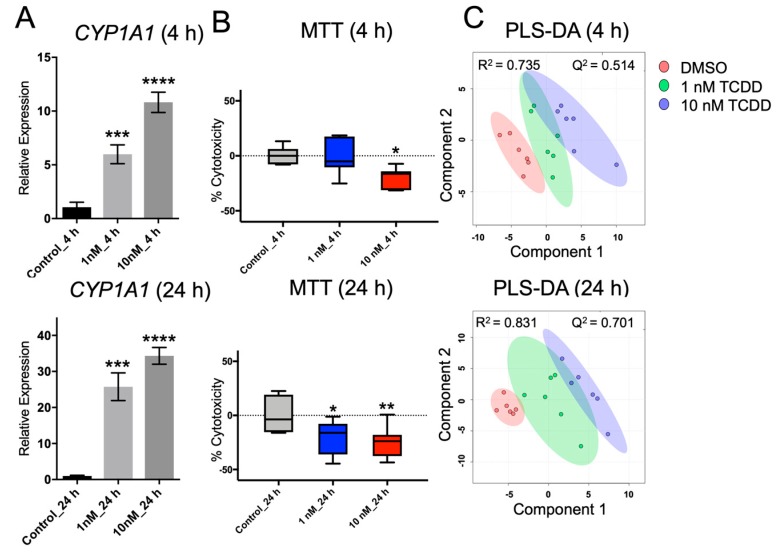
(**A**) Relative expression levels of *CYP1A1* normalized to *β-ACTIN* following 2,3,7,8-tetrachlorodibenzo-*p*-dioxin (TCDD) treatment (N = 6). (**B**) Percent cytotoxicity (% cytotoxicity) of each group by MTT assay (N = 3). (**C**) Scores plot of partial least squares discriminant analysis (PLS-DA) of polar metabolites in Huh-7 cells treated with different concentrations of TCDD. Each point represents a metabolite profile of a biological replicate (N = 6). A significant difference among groups was determined by one-way ANOVA with Dunnett’s multiple comparisons, *p*-value was adjusted for multiple comparisons. *, *p* < 0.05; **, *p* < 0.01; ***, *p* < 0.001; and ****, *p* < 0.0001.

Hepatoma cell lines are widely available and have been used extensively for liver metabolism studies [38,39,40,41,42]. TCDD is a representative ligand that activates the AHR [43]. *CYP1A1* expression was assessed to determine whether AHR was activated by TCDD. As shown in Figure 1A, *CYP1A1* expression was significantly increased in a dose-dependent manner in 1 nM and 10 nM of TCDD-treated Huh-7 cells. In this study, we aimed to find changes in the levels of polar metabolites using TCDD-exposed Huh-7 cells, which is a common in vitro toxicology model. Further, Huh-7 cells are a suitable model, as AHR is expressed and active [19,44,45].

Disruption of cell proliferation and apoptosis may lead to tumorigenicity, teratogenicity, and immunosuppression. Masayoshi Yamaguchi [37] reported that TCDD may suppress liver cancer cell (e.g., HepG2) growth through various signaling pathways, which are mediated by AHR and its related cofactors. However, TCDD has also been reported to prevent apoptosis in the MCF 10A cell line due to the inhibition of epidermal growth factor (EGF) [46,47]. Even though the underlying mechanisms remain unclear and require further investigation, sufficient evidence has revealed that TCDD is a potent tumor promoter in the liver and the skin of mice [48,49,50]. In this study, cell viability was determined by MTT assay (Figure 1B), and cytotoxicity of TCDD-treated cells decreased significantly compared with the untreated Huh-7 cells, indicating that the TCDD doses exploited in our study may increase metabolic activity, promote the proliferation, or prevent the apoptosis of Huh-7 cells. Protein quantification data also suggested that the cell number did not significantly change after TCDD treatment (Figure S6).

**Figure 2:**
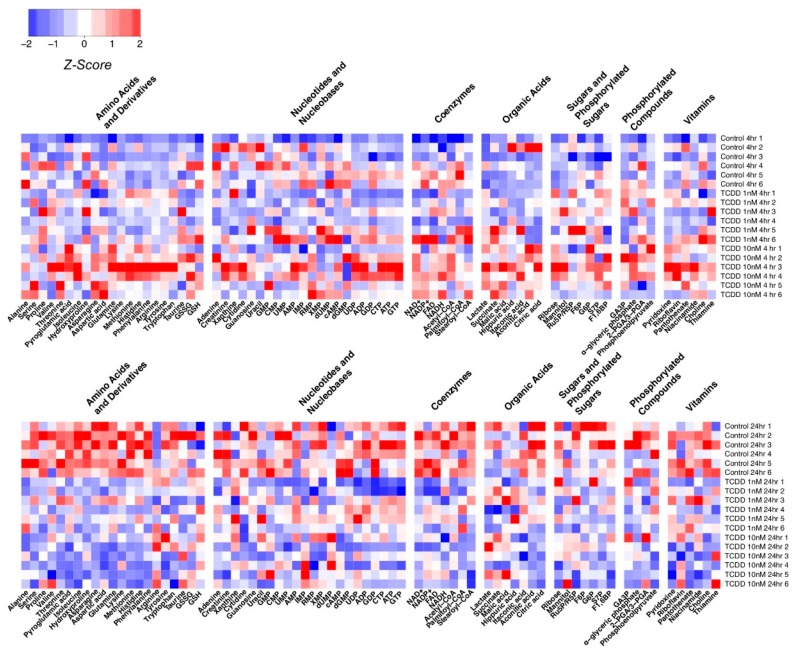
Quantitated polar metabolite patterns following exposure to TCDD (N = 6). The Z-score of each feature is plotted according to the red–blue color scale. The red and blue colors of the tile indicate high abundance and low abundance, respectively. Z-scores were created with the formula Z = ((*x* − $\bar{x}$))/(*sd*(*x*)), where *x* is the individual metabolite, $\bar{x}$ is the average of the metabolite across all the groups, and *sd*(*x*) is the standard deviation of each metabolite across all the groups. Z-scores were used to aid in data visualization through heatmaps. The Z-scores were imported into R, and the heatmap.2 command from the G plots package was used to create the heatmaps. The metabolites were not sorted, but were instead separated into groups based on metabolite category.

**Figure 3:**
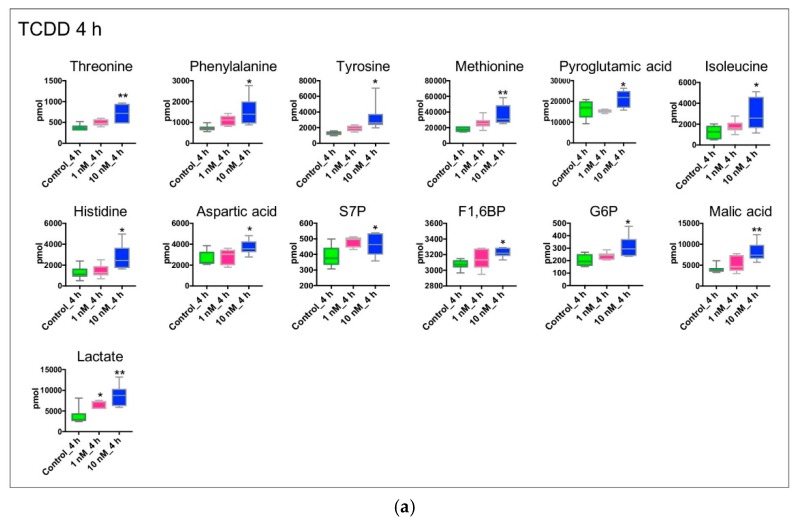

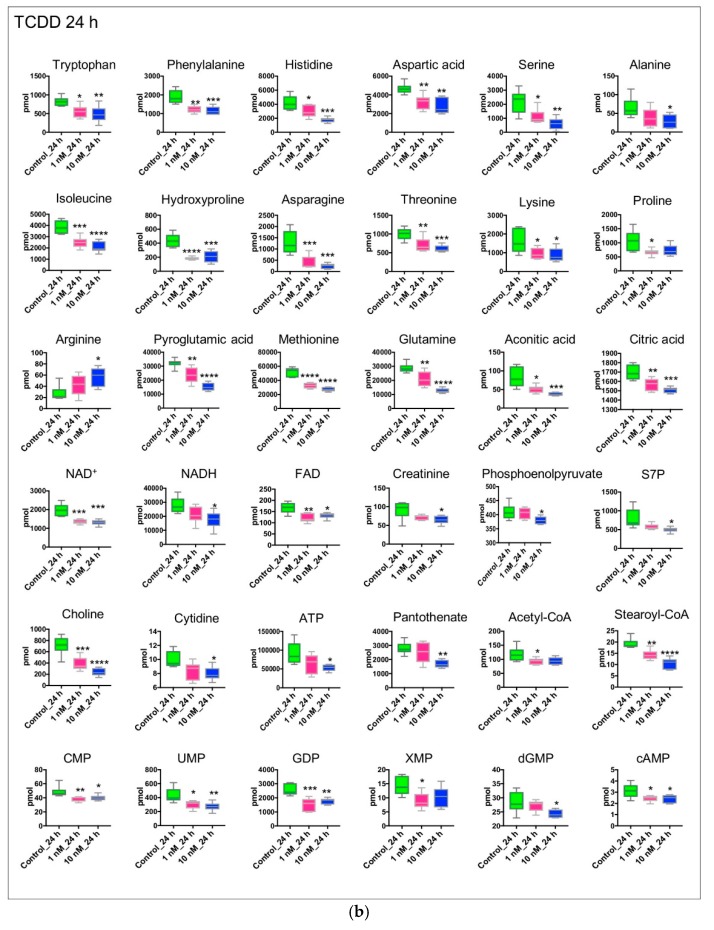
Concentration-dependent effects of TCDD on the metabolism of cultured Huh-7 cells (N = 6). Cells were incubated for 4 and 24 h with DMSO, 1 nM of TCDD, or 10 nM of TCDD. DMSO treated cells served as controls. For 4-h treatment, metabolites were distributed according to their roles in glycolytic metabolism (F1,6BP, G6P, lactate), TCA cycle (malic acid), PPP (S7P), amino acid metabolism (threonine, phenylalanine, isoleucine, etc). For 24-h treatment, metabolites were distributed according to their roles in energy metabolism (ATP, NADH, NAD^+^, FAD), glycolytic metabolism (phosphoenolpyruvate), TCA cycle (citric acid, aconitic acid), PPP (S7P), amino acid metabolism (e.g., glutamine, serine, alanine, leucine) and nucleic acid metabolism (e.g., CMP, UMP, GDP, XMP). Significant difference among groups was determined by one-way ANOVA with Dunnett’s multiple comparisons, *p*-value was adjusted for multiple comparisons. *, *p* < 0.05; **, *p* < 0.01; ***, *p* < 0.001; and ****, *p* < 0.0001. The cell number was approximately 1.00E+06 per sample. Metabolite concentration was measured by the HILIC–UHPLC–MS/MS method. (**a**) 4 h treatment. (**b**) 24 h treatment.

Figure 1C presents the PLS-DA plots of Huh-7 cells with 4 h and 24 h of TCDD treatment; the PCA plots are shown in Figure S7. Figure 2 shows the variation patterns of polar metabolites quantification in the presence of TCDD. The biological replicates of each group were clustered after both 4 h and 24 h of treatment, reflecting the difference in the magnitude of the effect of TCDD. For 4 h of TCDD treatment, the intermediates and products of glycolysis, F1,6BP, G6P, and lactate increased significantly, and amino acids (e.g., threonine, phenylalanine, isoleucine, and histidine) also increased (Figure 3), which may be a signal of protein degradation during apoptosis. The majority of changes and toxicity were observed at 24 h according to the PLS-DA, PCA results, and heatmap (Figure 2). Fold changes for the metabolites significantly affected by TCDD exposure are shown in Table S8. Most of the amino acids were downregulated with TCDD treatment at around 24 h, while arginine was increased. Arginine attenuated cell apoptosis [51], which is consistent with our MTT data. Arginine has been reported to enhance the production of NO and reduce liver cell necrosis and apoptosis by attenuating *B-cell lymphoma 2* gene expression in ischemia and reperfusion-induced injury [51,52]. These results indicate that TCDD at a low dose may enhance the production of arginine, the cells using nutrients for damage repair, or cell proliferation, so as to promote or maintain tumor cell growth. Several metabolites following 24 h of TCDD treatment were also detected by ^1^H NMR-based metabolomics (Figure S8, Figure S9). For example, most of the amino acid levels (leucine, isoleucine, valine, and aspartate) detected by ^1^H NMR were significantly depleted (or in the case of glutamine, showed marginal depletion, *p* = 0.077) as a result of 10 nM of TCDD treatment. These observations were highly consistent with the HILIC–UHPLC–MS/MS data.

**Figure 4:**
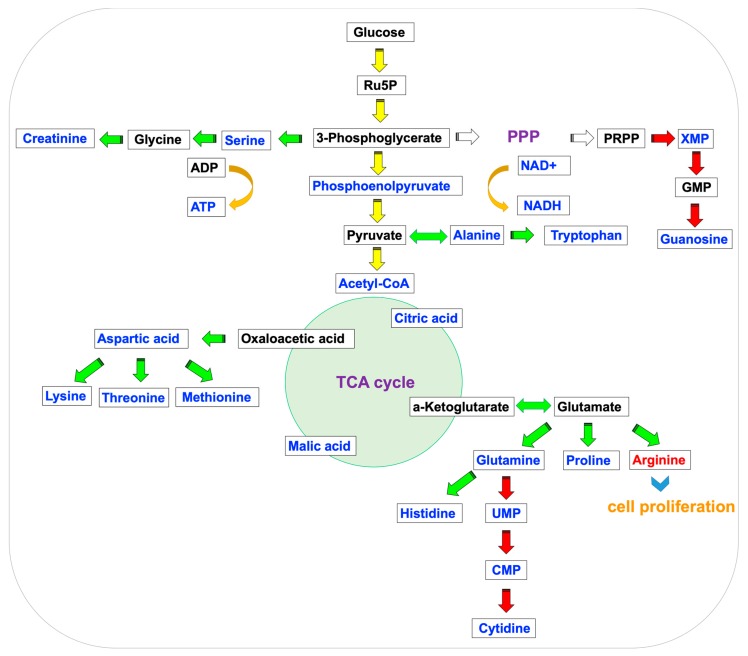
Illustration of the central metabolic pathway in 24-h TCDD-treated Huh-7 cells. The glycolytic pathway (yellow), amino acid metabolism (green), and nucleic acid metabolism (red) are shown. The red and blue color of the metabolites represent increasing or decreasing in the treatment group compared with the control group.

Dose-dependent ATP depletion was observed in the cells treated with TCDD at 1 nM and 10 nM for 24 h. After 24 h of incubation, intracellular ATP content was reduced to 69.9 ± 8.6% of the control group (*p* < 0.05), which may be related to mitochondrial dysfunction [36]. An overview of metabolite changes for 24 h of treatment is shown in Figure 4. Acetyl–CoA, citrate, NADH, and glycolytic intermediates, including phosphoenolpyruvate were decreased, indicating that energy metabolism (e.g., glycolysis, TCA cycle) is strongly affected. Amino acid and nucleic acid pools are reduced. These findings are supportive of the wasting syndrome [53], which is consistent with a recent study examining the metabolic impact of 10 nM of TCDD on HepG2 cells. In addition, in the ^1^H NMR data, lactate was depleted, thus indicating decreased glycolytic activity. Taurine levels were increased after TCDD exposure, which was consistent with the results reported for TCDD-exposed HepG2 cells [53]. Even though the mechanism for its increase remains unclear, taurine is involved in many metabolic pathways, including amino acid metabolism, fatty acid, and lipid metabolism and DNA repair [54].

Following 24 h of treatment, Huh-7 cells exhibited early signs of apoptosis, including amino acid metabolism dysfunction and alterations in glycolysis and the TCA cycle. While the MTT data suggested cell proliferation, this result may be due to a delay in the biological effect of TCDD exposure [55]. To clarify differences in cell viability and metabolite changes, additional doses and timepoints are warranted.

## 3. Conclusions

The HILIC–MS/MS method described here allows for the analysis and quantitation of 107 polar metabolites. This method permits polar metabolite detection and quantitation to study the effect of environmentally relevant toxicants such as TCDD at doses more clearly resembling those found in typical exposure scenarios. The results are comparable with previous data examining TCDD toxicity, which serves to validate the metabolomics results produced in this study.

## 4. Materials and Methods

### 4.1. Chemicals and Reagents

Polar metabolite standards, stable isotope standards [isoleucine (^13^C_6_, ^15^N), alanine (2,3-^13^C_2_), aspartic acid (U-^13^C_4_, ^15^N), glutamine (U-^13^C_5_, U-^15^N_2_), uracil (1,3-^15^N_2_), palmitoyl–CoA (^13^C_16_), AMP (^13^C_10_, ^15^N_5_), malic acid (^13^C_4_), succinic acid (1,4-^13^C_2_), and pyruvate (3-^13^C)] were purchased from Cambridge Isotope Laboratories (Tewksbury, MA, USA). Ammonium acetate (LC-MS grade) and 3-bromopyruvate (>99%) were purchased from Sigma (St. Louis, MO, USA). HPLC grade solvents were purchased from Fisher Scientific (Hampton, NH, USA).

### 4.2. HILIC-UHPLC-MS/MS Analysis

A Waters ACQUITY^TM^ Ultra Performance Liquid Chromatography system (Waters Corporation, Milford, MA, USA) coupled with a triple quadrupole mass spectrometer (Waters Xevo TQD, Milford, MA, USA) was used for UHPLC–MS/MS analysis. The MS was operated in both positive and negative mode. The instrument parameters were as follows: capillary voltage: 2500 V (positive mode) and 2000 V (negative mode); desolvation temperature: 450 °C; source temperature: 250 °C; cone gas flow: 150 L/h; and, desolvation gas flow: 1000 L/h.

MRM transitions, collision, and cone voltages were optimized for each metabolite by direct infusion into the MS with standard or internal standard solution at a combined flow rate of 20 μL/min in both positive and negative mode.

HILIC separation was achieved on an Acquity UHPLC BEH amide column (2.1 × 100 mm I.D., 1.7 μm) with solvent A (20 mM of ammonium acetate in 90% H_2_O/ACN, pH = 9) and solvent B (20 mM of ammonium acetate in 90% ACN/H_2_O, pH = 9). The column temperature was kept at 30 °C. A total of 5 μL was injected onto the column, and the autosampler was maintained at 10 °C. The gradient was: t = 0.0 min, flow = 0.15 mL/min, 100% B solvent; t = 0.5 min, flow = 0.15 mL/min, 100% B solvent; t = 1.0 min, flow = 0.15 mL/min, 80% B solvent; t = 8.0 min, flow = 0.30 mL/min, 70% B solvent; t = 10.0 min, flow = 0.35 mL/min, 30% B solvent; t = 11.0 min, flow = 0.35 mL/min, 0% B solvent; t = 14.0 min, flow = 0.35 mL/min, 0% B solvent; t = 14.1 min, flow = 0.40 mL/min, 100% B solvent; t = 23.50 min, flow = 0.40 mL/min, 100% B solvent; t = 23.7 min, flow = 0.15 mL/min, 100% B solvent; t = 25.0 min, flow = 0.15 mL/min, 100% B solvent.

Method validation was determined by examining sensitivity (limit of detection (LOD), limit of quantitation (LOQ), linear range (LR), and precision (intraday and interday relative standard deviation (%RSD)). LOQ and LOD were determined at S/N ratios of 3 and 10, respectively, on the Waters Xevo TQD. Calibration curves were built using 11 serial dilutions of polar metabolite standard mix solutions spiked with isotopically labeled internal standards at 5 µM and chlorpropamide at 1 µM. Intraday repeatability was determined by running the same “spike-in” sample five times a day within a 24-h interval. An interday precision measurement was performed on the same samples on five different days. The concentrations were quantified by isotope standards.

### 4.3. Cell Experiment

Huh-7 cells were cultivated in Dulbecco’s modified Eagle’s medium (DMEM; Invitrogen, Carlsbad, CA, USA) supplemented with 10% fetal bovine serum (FBS; Invitrogen, Carlsbad, CA, USA), 1% penicillin/streptomycin (Invitrogen, Carlsbad, CA, USA) and cultivated at 37 °C in 5% CO_2_. The cells were passaged with appropriate split ratios every two to three days and cell culture media was replaced every 24 h. 3-BrPy was added at 5 µM, 15 µM, 50 µM, and 100 µM; then, it was incubated for 24 h, mock-treated, and glucose deprivation samples served as negative and positive controls, respectively. In glucose deprivation, DMEM with reduced glucose concentration (8% of glucose concentration in the regular cell culture medium) was prepared by mixing glucose-free DMEM with regular DMEM [56]. In the TCDD toxicant experiment, TCDD was added at 1 nM and 10 nM and incubated for 4 h and 24 h.

After treatment, media in the six-well plates were removed, and the cells were washed twice with ice-cold 1 X PBS. Then, 500 µL of pre-cooled methanol (−20 °C) was added to quench all the cellular processes. The cells were scraped and stored at −80 °C in methanol suspension for extraction.

### 4.4. Quantitative Real-Time PCR

RNA was extracted from cells by TRIzol reagent (Invitrogen, Carlsbad, CA, USA). All the extracted RNA samples were diluted to 1 μg/μL using nuclease-free water. cDNA was synthesized using 1.0 μL of RNA and 4 μL of qScript cDNA Supermix (Quanta Biosciences, Gaitherburg, MD, USA) in 15 μL of nuclease-free water. Real-time PCR was performed using 1 μL of cDNA, 3.6 μL of nuclease-free water, and 0.2 μL of each forward and reverse primers. Gene-specific primers were used in each reaction; the sequences of the primers were as follows: *CYP1A1* sense 5′-GAC CAC AAC CAC CAA GAA C-3′, antisense 5′-AGC GAA GAA TAG GGA TGA AG-3′, and all the results were normalized to *β-ACTIN* (sense 5′-GGC ATA GAG GTC TTT ACG GAT CTC-3′, antisense 5′-TAT TGG CAA CGA GCG GTT CC-3′). qPCR assays were carried out on an ABI Prism 7900HT Fast Real-Time PCR sequence detection system (Applied Biosystems, Waltham, MA, USA) using SYBR Green PCR Master Mix and analyzed according to the ΔΔCT method.

### 4.5. Metabolite Extraction for LC-MS/MS

Each sample was spiked with a mixture of stable isotope standards. First, 500 µL of the ice-cold chloroform was added and homogenized (Bertin Technologies, Rockville, MD, USA) at 6500 rpm for two cycles of 30 s each with 1.0-mm diameter zirconia/silica beads (BioSpec, Bartlesville, OK, USA). Then, 250 µL each of water and chloroform were added and homogenized again, and then centrifuged at 18,800 g, 4 °C, for 10 min. The supernatant was collected, evaporated to dryness in a Savant Speed-Vac (Thermo Scientific, Waltham, MA, USA), and dissolved in 100 µL of acetonitrile/water (*v*:*v* = 1:1) solution with 1 µM of chlorpropamide. Samples were stored at −20 °C until analysis.

### 4.6. Metabolite Extraction for ^1^H NMR

Approximately 20 million Huh-7 cells were cultured and harvested from 150-mm dishes with the same method described before. First, 1 mL of pre-cooled methanol/water (*v*:*v* = 2:1) solution was used to quench all the cellular processes, the samples were then frozen–thawed three times with liquid nitrogen and a 37 °C water bath, followed by ultrasonication for 15 min and centrifuged at 3200 g (Eppendorf, Hamburg, Germany) for 10 min at 4 °C. The extraction was repeated twice and supernatants were combined, evaporated to dryness (Thermo Scientific, Waltham, MA, USA), and reconstituted into 600 µL of phosphate buffer (K_2_HPO_4_/NaH_2_PO_4_, 0.1 M, pH 7.4, 50% *v*/*v* D_2_O) containing 0.005% sodium 3-trimethylsilyl [2,2,3,3-d_4_] propionate (TSP-d_4_) as a chemical shift reference. Following centrifugation, 550 µL of the supernatant was transferred to NMR tubes and stored at 4 °C until analysis.

### 4.7. Data Analysis

UHPLC–MS/MS data were acquired and processed with MassLynx software (version 4.1; Waters, Miford, MA, USA). ^1^H NMR data were acquired on a Bruker Avance NEO III 600MHz spectrometer equipped with a Bruker inverse cryogenic probe (Bruker BioSpin, Rheinstetten, Germany). ^1^H NMR data analysis was processed with Chenomx suit (version 8.42; Chenomx, Edmonton, AB, Canada). Graphical illustrations and statistical analyses were performed using Prism GraphPad (version 7; GraphPad Software Inc., La Jolla, CA, USA). Significant differences among groups was determined by one-way ANOVA with Dunnett’s test, *p*-value was adjusted for multiple comparisons. Heatmaps were plotted using RStudio (heatmap.2 in G plots). Principal components analysis (PCA) was performed using Simca-P+ (version 13.0; Umetrics, Umeå, Sweden). Partial least squares discriminant analysis (PLS-DA) was performed using MetaboAnalyst 4.0. Statistical significance was defined as *p* < 0.05.

## Supplementary Materials

The following are available online at https://www.mdpi.com/2218-1989/9/6/118/s1, Table S1: Validation results for HILIC–UHPLC–MS/MS method, Table S2: Details of 10 isotope internal standards in HILIC–UHPLC–MS/MS method, Table S3: Details of LC gradients in optimization of HILIC condition, Table S4: Stock and working standard Solutions for HILIC–UPLC–MS/MS method, Table S5: Quantitation of polar metabolites in each sample treated by 3-BrPy with different concentration, Table S6: Quantitation of polar metabolites in each sample treated by TCDD with different concentration with 4 h and 24 h, Table S7: Metabolites significantly affected by TCDD exposure by 4 h treatment, Table S8: Metabolites significantly affected by TCDD exposure by 24-h treatment, Figure S1: Chromatography comparison for selected 12 metabolites in the method optimization, Figure S2: Separation of five pairs of isomers, Figure S3: Pharmacologic targeting of aerobic glycolysis in Huh-7 cells, Figure S4: MTT, PCA, heatmap analysis of polar metabolites in Huh-7 cells infected with different concentration of 3-BrPy, Figure S5: Concentration-dependent effects of 3-BrPy on metabolism of cultured Huh-7 cells, Figure S6: Protein concentration determination in Huh-7 Cells using the standard BCA Protein Assay, Figure S7: PCA plot of polar metabolites in Huh-7 cells infected with 1 and 10 nM of TCDD in 4 and 24 h treatment, Figure S8: PCA and PLS-DA plot of polar metabolites in Huh-7 cells infected with 1 and 10 nM of TCDD in 24 h treatment by ^1^H NMR, Figure S9: Concentration-dependent effects of TCDD on metabolism of cultured Huh-7 cells by ^1^H NMR.
