# Supplementary material for: A Quantitative HILIC–MS/MS Assay of the Metabolic Response of Huh-7 Cells Exposed to 2,3,7,8-Tetrachlorodibenzo-p-Dioxin
Source: Metabolites. 2019 Jun 20;9(6):118. doi: 10.3390/metabo9060118 (PMC6631636; doi:10.3390/metabo9060118)

# Supplementary Materials

## A Quantitative HILIC-MS/MS Assay of the Metabolic Response of Huh-7 Cells Exposed to 2,3,7,8-Tetrachlorodibenzo-*p*-Dioxin

Qing Liu, Jingwei Cai, Robert G. Nichols[2], Yuan Tian[2,3], Jintao Zhang[2], Philip B. Smith[4], Yan Wang, Chao Yan, and Andrew D. Patterson*.

**Table S1**: Validation results for HILIC-UHPLC-MS/MS method

**Table S2**: Details of ten isotope internal standards in HILIC-UHPLC-MS/MS method

**Table S3**: Details of LC gradients in optimization of HILIC condition

**Table S4**: Stock and working standard Solutions for HILIC-UPLC-MS/MS method

**Table S5**: Quantitation of polar metabolites in each sample treated by 3-BrPy with different concentration

**Table S6**: Quantitation of polar metabolites in each sample treated by TCDD with different concentration with 4 hours and 24 hours

**Table S7**: Metabolites significantly affected by TCDD exposure by 4 h treatment.

**Table S8**: Metabolites significantly affected by TCDD exposure by 24 h treatment

**Figure S1**: Chromatography comparison for selected 12 metabolites in the method optimization

**Figure S2**: Separation of five pairs of isomers

**Figure S3**: Pharmacologic targeting of aerobic glycolysis in Huh-7 cells

**Figure S4**: MTT, PCA, heatmap analysis of polar metabolites in Huh-7 cells infected with different concentration of 3-BrPy.

**Figure S5**: Concentration-dependent effects of 3-BrPy on metabolism of cultured Huh-7 cells.

**Figure S6**: Protein concentration determination in Huh-7 Cells using the standard BCA Protein Assay

**Figure S7**: PCA plot of polar metabolites in Huh-7 cells infected with 1 and 10 nM of TCDD in 4 and 24 h treatment.

**Figure S8**: PCA and PLS-DA plot of polar metabolites in Huh-7 cells infected with 1 and 10 nM of TCDD in 24 h treatment by $^{1}H$ NMR.

**Figure S9**: Concentration-dependent effects of TCDD on metabolism of cultured Huh-7 cells by $^{1}H$ NMR.

**Table S1**. Validation results (linearity, sensitivity and repeatability) and MRM transitions (MT) of HILIC-MS/MS method for 107 metabolites. Calibration curve: x = concentration μM, y = target peak area relative to internal standards. Linear range (LR), limit of detection (LOD) and limit of quantitation (LOQ) expressed as μM, interday (inter) and intraday (intra) repeatability of "spike-in" samples (5 μM A and 50 μM B polar metabolites mixture) were expressed as the relative standard deviation (%RSD).

| No. | Category | Compound* | RT (min) | Area RSD | | Calibration curve | $R^2$ | LOD | LOQ | LR | MT | Mode | Internal Standard |
|---|---|---|---|---|---|---|---|---|---|---|---|---|---|
| | | | | Inter | Intra | | | | | | | | |
| 1 | Amino Acid | 3-Guanidipropionic | 6.57 | 4.1 | 9.6 | y = 12.121x - 3.4325 | 0.996 | 0.20 | 0.60 | LOQ - 50 | 132.1 > 72.1 | ES+ | Glutamine (U-$^{13}C$, U-$^{15}N_2$) |
| 2 | Amino Acid | Phenylalanine | 5.22 | 7.1 | 9.7 | y = 0.0958x + 0.0013 | 0.992 | 0.03 | 0.30 | LOQ - 20 | 166.0 > 120.0 | ES+ | Isoleucine ($^{13}C_6$,$^{15}N$) |
| 3 | Amino Acid | Histidine | 7.04 | 5.6 | 11.8 | y = 1.0416x - 0.3014 | 0.989 | 0.03 | 0.15 | LOQ - 20 | 156.2 > 110.2 | ES+ | Isoleucine ($^{13}C_6$,$^{15}N$) |
| 4 | Amino Acid | Tryptophan | 5.10 | 10.5 | 14.4 | y = 0.0005x - 0.0067 | 1.000 | 1.00 | 3.00 | LOQ - 200 | 205.1 > 188.0 | ES+ | Isoleucine ($^{13}C_6$,$^{15}N$) |
| 5 | Amino Acid | Isoleucine | 5.45 | 2.7 | 4.5 | y = 0.5114x + 0.0257 | 0.999 | 0.07 | 0.21 | LOQ - 20 | 132.1 > 43.3 | ES+ | Isoleucine ($^{13}C_6$,$^{15}N$) |
| 6 | Amino Acid | leucine | 6.53 | 7.1 | 10.7 | y = 20.363x + 0.4083 | 0.995 | 0.10 | 0.30 | LOQ - 20 | 132.1 > 86.0 | ES+ | Alanine (2,3-$^{13}C_2$) |
| 7 | Amino Acid | Asparagine | 6.92 | 7.0 | 17.0 | y = 0.4603x - 0.19 | 0.998 | 0.10 | 0.30 | LOQ - 20 | 133.2 > 87.2 | ES+ | Alanine (2,3-13$C_2$) |
| 8 | Amino Acid | Hydroxyproline | 6.49 | 3.9 | 16.1 | y = 0.1758x - 0.0452 | 0.997 | 0.33 | 0.10 | LOQ - 20 | 132.1 > 68.0 | ES+ | Glutamine (U-$^{13}C$, U-$^{15}N_2$) |
| 9 | Amino Acid | Serine | 6.96 | 9.5 | 19.3 | y = 0.4185x - 0.412 | 0.998 | 0.10 | 0.30 | LOQ - 20 | 106.1 > 60.0 | ES+ | Alanine (2,3-13$C_2$) |
| 10 | Amino Acid | Glutamine | 6.85 | 9.2 | 12.2 | y = 2.5097x - 0.7179 | 0.997 | 0.03 | 0.09 | LOQ - 20 | 147.1 > 84.01 | ES+ | Glutamine (U-$^{13}C$, U-$^{15}N_2$) |
| 11 | Amino Acid | Proline | 6.19 | 9.8 | 14.5 | y = 0.5755x + 0.1133 | 0.997 | 0.02 | 0.06 | LOQ - 10 | 116.0 > 70.0 | ES+ | Isoleucine ($^{13}C_6$,$^{15}N$) |
| 12 | Amino Acid | Alanine | 6.49 | 13.1 | 18.9 | y = 1.3002x + 0.0611 | 0.999 | 0.08 | 0.24 | LOQ - 20 | 90.1 > 44.0 | ES+ | Alanine (2,3-$^{13}C_2$) |
| 13 | Amino Acid | Arginine | 9.09 | 8.1 | 14.0 | y = 4.1759x - 1.7646 | 0.994 | 0.05 | 0.15 | LOQ - 20 | 175.2 > 70.2 | ES+ | Isoleucine ($^{13}C_6$,$^{15}N$) |
| 14 | Amino Acid | Threonine/Homoserine | 6.69 | 9.4 | 13.1 | y = 0.0971x + 0.0668 | 0.993 | 0.10 | 0.30 | LOQ - 20 | 120.1 > 74.0 | ES+ | Isoleucine ($^{13}C_6$,$^{15}N$) |

| | | | | | | | | | | | | | |
|---|---|---|---|---|---|---|---|---|---|---|---|---|---|
| 15 | Amino Acid | Ornithine | 4.83 | 9.9 | 12.1 | y = 0.3646x + 0.0732 | 0.995 | 0.15 | 0.45 | LOQ - 20 | 133.1>70.1 | ES+ | Alanine (2,3-$^{13}C_2$) |
| 16 | Amino Acid | Tyrosine | 5.79 | 8.4 | 10.8 | y = 0.0557x - 0.0156 | 0.995 | 0.30 | 1.00 | LOQ - 20 | 182.0 > 165.0 | ES+ | Aspartic acid (U-$^{13}C_4$,$^{15}N$) |
| 17 | Amino Acid | Lysine | 9.56 | 7.2 | 11.8 | y = 0.0909x - 0.0267 | 0.996 | 0.10 | 0.30 | LOQ - 20 | 147.2 > 84.2 | ES+ | Isoleucine ($^{13}C_6$,$^{15}N$) |
| 18 | Amino Acid | Glycine | 6.68 | 12.6 | 29.2 | y = 0.0221x - 0.0213 | 0.994 | 1.00 | 3.00 | LOQ - 200 | 76.1 > 48.0 | ES+ | Alanine (2,3-$^{13}C_2$) |
| 19 | Amino Acid | Aspartic acid | 7.65 | 5.2 | 9.2 | y = 0.0096x - 0.0033 | 0.990 | 0.10 | 0.30 | LOQ - 20 | 134.1 > 88.1 | ES+ | Isoleucine ($^{13}C_6$,$^{15}N$) |
| 20 | Amino Acid | Methionine | 5.61 | 8.0 | 11.4 | y = 0.3657x - 0.0297 | 0.998 | 0.10 | 0.30 | LOQ - 20 | 150.2 > 104.2 | ES+ | Aspartic acid (U-$^{13}C_4$,$^{15}N$) |
| 21 | Amino Acid | Pyroglutamic acid | 6.80 | 6.8 | 9.8 | y = 1.8862x - 0.9517 | 0.998 | 0.01 | 0.03 | LOQ - 20 | 130.1 > 84.0 | ES+ | Alanine (2,3-13$C_2$) |
| 22 | Amino Acid | Theanine | 5.82 | 4.2 | 9.4 | y = 4.1865x - 1.1609 | 0.997 | 0.02 | 0.06 | LOQ - 20 | 175.2 > 46.1 | ES+ | Alanine (2,3-13$C_2$) |
| 23 | Amino Acid | Valine | 5.88 | 4.1 | 8.7 | y = 0.1562x + 0.021 | 0.994 | 0.10 | 0.30 | LOQ - 20 | 118.1 > 72.0 | ES+ | Isoleucine ($^{13}C_6$,$^{15}N$) |
| 24 | Amino Acid derivative | Taurine | 6.11 | 8.4 | 15.8 | y = 0.2052x - 0.2205 | 0.992 | 0.20 | 0.60 | LOQ - 20 | 126.1 > 108.0 | ES+ | Alanine (2,3-13$C_2$) |
| 25 | Amino Acid derivative | GSSG | 7.62 | 8.6 | 14.3 | y = 0.0482x - 0.0059 | 0.994 | 0.15 | 0.45 | LOQ - 20 | 613.1 > 355.1 | ES+ | AMP ($^{13}C_{10}$,$^{15}N_5$) |
| 26 | Amino Acid derivative | GSH | 7.62 | 10.6 | 26.4 | y = 0.0039x - 0.0076 | 0.992 | 0.10 | 0.30 | LOQ - 200 | 308.2 > 179.1 | ES+ | Isoleucine ($^{13}C_6$,$^{15}N$) |
| 27 | Coenzyme | Acetyl CoA | 8.12 | 6.4 | 12.0 | y = 0.1422x - 0.0073 | 1.000 | 0.01 | 0.03 | LOQ - 20 | 810.2 > 303.2 | ES+ | Isoleucine ($^{13}C_6$,$^{15}N$) |
| 28 | Coenzyme | $NADP^+$ | 10.23 | 5.6 | 11.6 | y = 0.9124x + 0.0148 | 0.997 | 0.01 | 0.03 | LOQ - 20 | 744.1 > 136.1 | ES+ | Isoleucine ($^{13}C_6$,$^{15}N$) |
| 29 | Coenzyme | NADPH | 9.59 | 10.9 | 14.4 | y = 1E-04x - 0.0009 | 0.992 | 2.00 | 6.00 | LOQ - 200 | 744.0 > 426.1 | ES- | Succinate ($^{13}C$) |
| 30 | Coenzyme | $NAD^+$ | 8.30 | 6.4 | 9.0 | y = 0.3097x - 0.091 | 0.992 | 0.01 | 0.03 | LOQ - 20 | 664.2 > 136.1 | ES+ | Isoleucine ($^{13}C_6$,$^{15}N$) |
| 31 | Coenzyme | NADH | 7.55 | 8.6 | 14.7 | y = 0.0096x - 0.0026 | 0.995 | 0.50 | 1.50 | LOQ - 20 | 666.1 > 649.1 | ES+ | Isoleucine ($^{13}C_6$,$^{15}N$) |
| 32 | Coenzyme | FAD | 7.39 | 7.8 | 12.1 | y = 0.1071x - 0.0069 | 0.996 | 0.10 | 0.30 | LOQ - 200 | 786.2 > 136.1 | ES+ | Isoleucine ($^{13}C_6$,$^{15}N$) |
| 33 | Coenzyme | Palmitoyl CoA | 4.85 | 10.8 | 19.9 | y = 0.0686x - 0.0001 | 0.998 | 0.03 | 0.10 | LOQ - 20 | 1006.5 >499.4 | ES+ | Palmitoyl-CoA ($^{13}C_{16}$) |
| 34 | Nucleotide | cAMP | 5.71 | 6.9 | 10.1 | = 0.1982x + 0.0025 | 0.995 | 0.07 | 0.20 | LOQ - 20 | 330.2 > 136.1 | ES+ | AMP ($^{13}C_{10}$,$^{15}N_5$) |

| | | | | | | | | | | | | | |
|---|---|---|---|---|---|---|---|---|---|---|---|---|---|
| 35 | Nucleotide | dUMP | 8.03 | 6.8 | 14.3 | y = 0.0019x + 0.0158 | 0.997 | 0.30 | 1.00 | LOQ - 20 | 309.1 > 81.0 | ES+ | AMP ($^{13}C_{10}$,$^{15}N_5$) |
| 36 | Nucleotide | dCMP | 8.62 | 7.6 | 12.0 | y = 0.0151x - 0.0296 | 0.996 | 0.10 | 0.30 | LOQ - 20 | 308.2 > 112.0 | ES+ | Isoleucine ($^{13}C_6$,$^{15}N$) |
| 37 | Nucleotide | cGMP | 6.16 | 8.2 | 13.5 | y = 0.073x - 5E-05 | 0.993 | 0.05 | 0.15 | LOQ - 20 | 346.1 > 152.0 | ES+ | Isoleucine ($^{13}C_6$,$^{15}N$) |
| 38 | Nucleotide | dGMP | 8.77 | 6.7 | 11.0 | y = 0.0458x + 0.0214 | 0.993 | 0.03 | 1.00 | LOQ - 20 | 348.1 > 152.0 | ES+ | AMP ($^{13}C_{10}$,$^{15}N_5$) |
| 39 | Nucleotide | ADP | 9.20 | 5.1 | 12.7 | y = 0.1282x - 0.0088 | 0.998 | 0.02 | 0.06 | LOQ - 20 | 428.1 > 136.1 | ES+ | AMP ($^{13}C_{10}$,$^{15}N_5$) |
| 40 | Nucleotide | GDP | 10.07 | 4.8 | 9.4 | y = 0.0599x - 0.0026 | 0.999 | 0.10 | 0.30 | LOQ - 20 | 444.1 > 152.0 | ES+ | AMP (13$C_{10}$,$^{15}N_6$) |
| 41 | Nucleotide | UDP | 9.63 | 7.3 | 12.4 | y = 0.0694x - 0.0236 | 0.997 | 0.10 | 0.30 | LOQ - 20 | 405.1 > 97.0 | ES+ | AMP ($^{13}C_{10}$,$^{15}N_5$) |
| 42 | Nucleotide | ATP | 10.23 | 3.1 | 7.1 | y = 0.0703x - 0.0034 | 0.994 | 0.02 | 0.06 | LOQ - 20 | 508.1 > 136.1 | ES+ | AMP ($^{13}C_{10}$,$^{15}N_5$) |
| 43 | Nucleotide | CTP | 10.35 | 8.1 | 10.1 | y = 0.0789x - 0.0274 | 0.993 | 0.15 | 0.45 | LOQ - 20 | 484.0 > 112.0 | ES+ | AMP ($^{13}C_{10}$,$^{15}N_5$) |
| 44 | Nucleotide | GTP | 10.42 | 4.7 | 9.8 | y = 0.0525x - 0.0031 | 0.998 | 0.20 | 0.60 | LOQ - 20 | 524.0 > 152.1 | ES+ | AMP ($^{13}C_{10}$,$^{15}N_5$) |
| 45 | Nucleotide | TMP | 7.79 | 14.0 | 14.3 | y = 0.048x + 0.0051 | 0.996 | 0.07 | 0.20 | LOQ - 20 | 323.1 > 81.0 | ES+ | Isoleucine ($^{13}C_6$,$^{15}N$) |
| 46 | Nucleotide | UMP | 8.66 | 3.7 | 6.4 | y = 0.0855x - 0.0091 | 0.997 | 0.05 | 0.15 | LOQ - 20 | 325.2 > 97.0 | ES+ | Isoleucine ($^{13}C_6$,$^{15}N$) |
| 47 | Nucleotide | CMP | 9.13 | 3.7 | 11.2 | y = 0.177x - 0.0948 | 0.990 | 0.07 | 0.20 | LOQ - 20 | 324.1 > 112.04 | ES+ | Isoleucine ($^{13}C_6$,$^{15}N$) |
| 48 | Nucleotide | GMP | 8.85 | 5.4 | 14.5 | y = 0.0007x - 0.0101 | 1.000 | 2.00 | 6.00 | LOQ - 20 | 362.1 > 211.0 | ES- | AMP ($^{13}C_{10}$,$^{15}N_5$) |
| 49 | Nucleotide | AMP | 8.22 | 2.5 | 9.4 | y = 0.1643x + 0.0217 | 0.998 | 0.02 | 0.06 | LOQ - 20 | 348.2 > 136.0 | ES+ | AMP ($^{13}C_{10}$,$^{15}N_5$) |
| 50 | Nucleotide | XMP | 9.23 | 6.1 | 14.6 | y = 0.0048x + 0.0039 | 0.991 | 0.15 | 0.45 | LOQ - 20 | 365.1 > 153.1 | ES+ | AMP ($^{13}C_{10}$,$^{15}N_5$) |
| 51 | Nucleotide | IMP | 8.81 | 9.6 | 10.5 | y = 0.0727x - 0.0061 | 0.999 | 0.03 | 0.09 | LOQ - 20 | 349.1 > 137.1 | ES+ | AMP ($^{13}C_{10}$,$^{15}N_5$) |
| 52 | Nucleotide | RMP | 7.55 | 9.1 | 12.3 | y = 0.0423x + 0.0037 | 0.998 | 0.01 | 0.03 | LOQ - 20 | 457.2 > 439.1 | ES+ | AMP ($^{13}C_{10}$,$^{15}N_5$) |
| 53 | Nucleobase | Creatinine | 4.83 | 5.0 | 13.4 | y = 1.8262x + 0.0374 | 1.000 | 0.03 | 0.10 | LOQ - 20 | 114.1 > 114.1 | ES+ | Isoleucine ($^{13}C_6$,$^{15}N$) |
| 54 | Nucleobase | Inosine | 5.37 | 5.4 | 14.3 | y = 0.0997x - 0.0275 | 0.995 | 0.10 | 0.30 | LOQ - 20 | 269.1 > 137.0 | ES+ | Isoleucine ($^{13}C_6$,$^{15}N$) |

| | | | | | | | | | | | | | |
|---|---|---|---|---|---|---|---|---|---|---|---|---|---|
| 55 | Nucleobase | Guanine | 5.58 | 10.6 | 16.0 | y = 0.3605x + 0.18 | 0.998 | 0.33 | 1.00 | LOQ - 20 | 152.1 > 135.0 | ES+ | Aspartic acid (U-$^{13}C_4$,$^{15}N$) |
| 56 | Nucleobase | Uridine | 4.91 | 14.6 | 17.2 | y = 0.0252x - 0.0664 | 0.998 | 0.30 | 1.00 | LOQ - 20 | 245.2 > 113.0 | ES+ | Isoleucine ($^{13}C_6$,$^{15}N$) |
| 57 | Nucleobase | Xanthine | 5.47 | 4.8 | 7.8 | y = 0.1449x + 0.1043 | 0.992 | 0.39 | 1.20 | LOQ - 25 | 151.0 > 108.0 | ES- | Uracil (1,3-$^{15}N_2$) |
| 58 | Nucleobase | Thymine | 2.62 | 8.5 | 13.2 | y = 0.0011x - 0.0018 | 0.997 | 0.13 | 0.39 | LOQ - 20 | 127.1 > 54.1 | ES+ | Isoleucine ($^{13}C_6$,$^{15}N$) |
| 59 | Nucleobase | Uracil | 3.04 | 4.7 | 8.1 | y = 0.0309x - 0.046 | 0.998 | 0.10 | 0.30 | LOQ - 200 | 111.1 > 42.0 | ES- | Uracil (1,3-$^{15}N_2$) |
| 60 | Nucleobase | Hypoxanthine | 5.37 | 7.5 | 15.0 | y = 0.0162x - 0.0143 | 0.993 | 0.02 | 0.60 | LOQ - 20 | 137.1 > 119.0 | ES+ | Isoleucine ($^{13}C_6$,$^{15}N$) |
| 61 | Nucleobase | Adenine | 4.50 | 4.3 | 6.0 | y = 12.38x - 4.0948 | 0.998 | 0.01 | 0.03 | LOQ - 20 | 136.1 > 119.0 | ES+ | Isoleucine ($^{13}C_6$,$^{15}N$) |
| 62 | Nucleobase | Guanosine | 5.58 | 7.3 | 9.6 | y = 0.1567x - 0.0122 | 0.995 | 0.07 | 0.20 | LOQ - 20 | 284.2 > 152.1 | ES+ | Isoleucine ($^{13}C_6$,$^{15}N$) |
| 63 | Nucleobase | Cytidine | 5.52 | 10.1 | 14.7 | y = 0.2119x - 0.0226 | 0.999 | 0.03 | 0.10 | LOQ - 20 | 244.1 > 112.0 | ES+ | Isoleucine ($^{13}C_6$,$^{15}N$) |
| 64 | Nucleobase | Cytosine | 5.06 | 6.5 | 22.3 | y = 0.1207x - 0.1394 | 0.992 | 0.70 | 2.10 | LOQ - 20 | 112.1 > 69.0 | ES+ | Alanine (2,3-13$C_2$) |
| 65 | Organic Acid | Itaconic acid | 6.79 | 5.1 | 9.9 | y = 0.019x + 0.1103 | 0.994 | 0.50 | 1.50 | LOQ - 100 | 129.0 > 85.0 | ES- | Succinate (1,4-$^{13}C_2$) |
| 66 | Organic Acid | α-ketoglutaric acid | 5.15 | 6.0 | 7.5 | y = 0.0338x - 0.0083 | 0.999 | 0.33 | 0.10 | LOQ - 20 | 147.1 > 119.1 | ES+ | Isoleucine ($^{13}C_6$,$^{15}N$) |
| 67 | Organic Acid | Fumarate | 6.76 | 6.6 | 9.4 | y = 0.0047x + 0.0037 | 0.998 | 0.39 | 1.20 | LOQ - 50 | 115.0 > 70.0 | ES- | Succinate (1,4-$^{13}C_2$) |
| 68 | Organic Acid | Hippuric acid | 4.82 | 4.5 | 5.0 | y = 0.0308x + 0.0308 | 0.996 | 0.25 | 0.75 | LOQ - 200 | 178.1 > 134.0 | ES- | Succinate (1,4-$^{13}C_2$) |
| 69 | Organic Acid | Malic acid | 7.34 | 2.5 | 3.3 | y = 0.1871x + 0.2564 | 0.995 | 0.25 | 0.75 | LOQ - 200 | 133.0 > 115.0 | ES- | Malic acid ($^{13}C_4$) |
| 70 | Organic Acid | Oritic acid | 5.53 | 4.1 | 4.8 | y = 0.1577x + 0.1494 | 0.990 | 0.08 | 0.24 | LOQ - 25 | 155.1 > 111.0 | ES- | Malic acid ($^{13}C_4$) |
| 71 | Organic Acid | Lactate | 5.68 | 2.2 | 8.0 | y = 0.0235x - 0.021 | 0.999 | 0.20 | 0.60 | LOQ - 200 | 89.0 > 89.5 | ES- | Succinate (1,4-$^{13}C_2$) |
| 72 | Organic Acid | Aconitic acid | 8.24 | 7.7 | 14.5 | y = 0.063x - 0.2488 | 0.997 | 1.50 | 4.50 | LOQ - 200 | 173.1 > 129.0 | ES- | Malic acid ($^{13}C_4$) |
| 73 | Organic Acid | Shikimic acid | 6.54 | 6.9 | 12.3 | y = 0.0035x + 0.0059 | 0.998 | 3.00 | 9.00 | LOQ - 200 | 173.0 > 93.0 | ES- | Succinate (1,4-$^{13}C_2$) |
| 74 | Organic Acid | Succinate | 7.06 | 2.1 | 4.7 | y = 0.0265x + 0.1029 | 0.996 | 0.20 | 0.60 | LOQ - 200 | 117.1 > 73.1 | ES- | Succinate (1,4-$^{13}C_2$) |

| | | | | | | | | | | | | | |
|---|---|---|---|---|---|---|---|---|---|---|---|---|---|
| 75 | Organic Acid | Pyruvate | 4.90 | 12.7 | 15.7 | y = 0.0065x - 0.0022 | 0.994 | 0.39 | 1.20 | LOQ - 200 | 87.0 > 43.0 | ES- | Succinate (1,4-$^{13}C_2$) |
| 76 | Organic Acid | Glyceric acid | 6.32 | 10.0 | 14.6 | y = 0.0129x + 0.0614 | 0.991 | 0.20 | 0.60 | LOQ - 200 | 105.0 > 75.0 | ES- | Malic acid ($^{13}C_4$) |
| 77 | Organic Acid | Citrate/Isocitrate | 8.84 | 4.1 | 12.3 | y = 0.0923x - 1.9041 | 0.999 | 1.56 | 4.68 | LOQ - 200 | 191.1 > 111.0 | ES- | Succinate (1,4-$^{13}C_2$) |
| 78 | Phosphorylated compound | DHAP | 7.96 | 5.2 | 9.6 | y = 0.0066x - 0.0258 | 0.998 | 0.10 | 0.30 | LOQ - 200 | 169.0 > 79.0 | ES- | Malic acid ($^{13}C_4$) |
| 79 | Phosphorylated compound | Phosphocreatine | 8.69 | 8.1 | 23.8 | y = 0.0004x + 0.0366 | 0.996 | 0.30 | 1.50 | LOQ - 200 | 212.1 > 90.0 | ES+ | Aspartic acid (U-$^{13}C_4$,$^{15}N$) |
| 80 | Phosphorylated compound | β-glyceric phosphate | 7.62 | 5.4 | 12.6 | y = 0.0758x - 0.0822 | 0.997 | 0.20 | 0.60 | LOQ - 200 | 173.1 > 99.0 | ES+ | Isoleucine ($^{13}C_6$,$^{15}N$) |
| 81 | Phosphorylated compound | α-glyceric phosphate | 7.95 | 11.8 | 18.1 | y = 0.0452x - 0.1338 | 0.993 | 1.0 | 3.0 | LOQ - 200 | 173.1 > 99.0 | ES+ | Isoleucine ($^{13}C_6$,$^{15}N$) |
| 82 | Phosphorylated compound | KDPG | 8.91 | 5.2 | 9.2 | y = 0.0081x - 0.1144 | 0.999 | 3.30 | 10.00 | LOQ - 200 | 257.1 > 97.0 | ES- | Succinate (1,4-$^{13}C_2$) |
| 83 | Phosphorylated compound | GA3P | 8.84 | 5.1 | 9.7 | y = 0.0055x - 0.0061 | 0.992 | 0.50 | 1.50 | LOQ - 100 | 171.1 > 99.0 | ES+ | AMP ($^{13}C_{10}$,$^{15}N_5$) |
| 84 | Phosphorylated compound | 2-PGA/3-PGA | 6.01 | 3.7 | 10.2 | y = 0.0089x + 1.0084 | 0.998 | 1.00 | 3.00 | LOQ - 200 | 187.1 > 105.0 | ES+ | AMP ($^{13}C_{10}$,$^{15}N_5$) |
| 85 | Phosphorylated compound | Phosphoenolpyruvate | 8.69 | 6.3 | 14.8 | y = 0.0092x - 0.0236 | 0.996 | 1.50 | 4.50 | LOQ - 25 | 169.0 > 151.0 | ES+ | Isoleucine ($^{13}C_6$,$^{15}N$) |
| 86 | Phosphorylated compound | 6-Phosphogluconate | 9.71 | 5.4 | 6.0 | y = 0.0082x - 0.123 | 0.993 | 1.56 | 4.68 | LOQ - 200 | 275.0 > 97.0 | ES- | Succinate (1,4-$^{13}C_2$) |
| 87 | Phosphorylated compound | E4P | 8.88 | 10.5 | 14.1 | y = 0.0006x + 0.0011 | 0.992 | 7.00 | 21.00 | LOQ - 200 | 201.0 > 103.0 | ES+ | Isoleucine ($^{13}C_6$,$^{15}N$) |
| 88 | Phosphorylated sugar | R5P | 8.45 | 4.6 | 7.0 | y = 0.0096x - 0.0511 | 0.995 | 2.00 | 6.00 | LOQ - 200 | 229.1 > 97.0 | ES+ | AMP ($^{13}C_{10}$,$^{15}N_5$) |
| 89 | Phosphorylated sugar | Ru5P | 8.43 | 10.5 | 14.3 | y = 0.0128x - 0.0035 | 0.992 | 2.00 | 6.00 | LOQ - 200 | 229.1 > 97.0 | ES- | AMP ($^{13}C_{10}$,$^{15}N_5$) |
| 90 | Phosphorylated sugar | F1,6BP | 10.44 | 9.8 | 11.7 | y = 0.0044x - 0.0229 | 0.990 | 1.00 | 3.00 | LOQ - 200 | 341.1 > 109.0 | ES+ | Isoleucine ($^{13}C_6$,$^{15}N$) |
| 91 | Phosphorylated sugar | R1,5BP | 10.40 | 12.5 | 14.3 | y = 0.2172x + 0.0327 | 0.993 | 0.20 | 0.60 | LOQ - 200 | 311.1 > 99.2 | ES+ | Isoleucine ($^{13}C_6$,$^{15}N$) |
| 92 | Phosphorylated sugar | G6P | 9.20 | 3.3 | 9.1 | y = 0.0315x - 0.2521 | 0.991 | 0.78 | 2.34 | LOQ - 200 | 259.1 > 97.0 | ES- | Uracil (1,3-$^{15}N_2$) |
| 93 | Phosphorylated sugar | F6P | 8.63 | 2.1 | 3.4 | y = 0.0249x - 0.1903 | 0.996 | 0.78 | 2.34 | LOQ - 200 | 259.0 > 97.0 | ES- | Uracil (1,3-$^{15}N_2$) |
| 94 | Phosphorylated sugar | S7P | 8.84 | 10.5 | 14.3 | y = 0.0074x + 0.0035 | 0.992 | 0.39 | 1.20 | LOQ - 200 | 313.1 > 295.0 | ES+ | Isoleucine ($^{13}C_6$,$^{15}N$) |

| | | | | | | | | | | | | | |
|---|---|---|---|---|---|---|---|---|---|---|---|---|---|
| 95 | Sugar | Mannitol | 6.15 | 5.0 | 7.5 | y = 0.0054x + 0.0043 | 1.000 | 0.13 | 0.39 | LOQ - 200 | 181.2 > 89.0 | ES- | Succinate (1,4-$^{13}C_2$) |
| 96 | Sugar | Ribose | 5.18 | 9.6 | 16.3 | y = 0.0002x - 0.0029 | 0.994 | 0.30 | 1.00 | LOQ - 200 | 149.1 > 89.0 | ES- | Succinate (1,4-$^{13}C_2$) |
| 97 | Vitamin | Folic acid | 8.52 | 4.8 | 7.5 | y = 0.007x - 0.0031 | 0.999 | 0.33 | 1.00 | LOQ - 200 | 440.2 > 311.2 | ES+ | AMP ($^{13}C_{10}$,$^{15}N_5$) |
| 98 | Vitamin | Pyridoxine | 3.50 | 7.0 | 11.5 | y = 0.1982x - 0.6633 | 0.998 | 0.15 | 0.45 | LOQ - 200 | 170.2 > 152.0 | ES+ | Isoleucine ($^{13}C_6$,$^{15}N$) |
| 99 | Vitamin | Pantothenate | 5.76 | 5.8 | 8.2 | y = 0.0089x - 0.0171 | 0.995 | 0.78 | 2.34 | LOQ - 50 | 220.1 > 90.1 | ES+ | Isoleucine ($^{13}C_6$,$^{15}N$) |
| 100 | Vitamin | Thiamine | 5.52 | 17.2 | 19.0 | y = 0.3104x - 0.0619 | 0.999 | 0.10 | 0.30 | LOQ - 20 | 267.2 > 122.6 | ES+ | Alanine (2,3-$^{13}C_2$) |
| 101 | Vitamin | Riboflavin | 4.85 | 5.1 | 10.2 | y = 0.084x + 0.0115 | 0.998 | 0.02 | 0.06 | LOQ - 20 | 377.2 > 243.2 | ES+ | AMP ($^{13}C_{10}$,$^{15}N_5$) |
| 102 | Vitamin | Choline | 5.30 | 12.2 | 19.8 | y = 17.15x - 0.3093 | 0.998 | 0.03 | 0.10 | LOQ - 20 | 104.1 > 60.1 | ES+ | Alanine (2,3-$^{13}C_2$) |
| 103 | Vitamin | Vit $B_{12}$ | 6.44 | 9.6 | 30.6 | y = 0.0085x - 0.0191 | 0.995 | 1.00 | 3.00 | LOQ - 20 | 1355.6 > 1209.6 | ES+ | Alanine (2,3-$^{13}C_2$) |
| 104 | Vitamin | Niacinamide | 2.47 | 6.6 | 12.2 | y = 0.9428x + 0.2706 | 0.998 | 0.01 | 0.03 | LOQ - 20 | 123.0 > 80.6 | ES+ | Isoleucine ($^{13}C_6$,$^{15}N$) |

* **GSSG**: Glutathione oxidized, **GSH**: Glutathione reduced, **NADP⁺**: *β*-Nicotinamide adenine dinucleotide phosphate oxidized, **NADPH**: *β*-Nicotinamide adenine dinucleotide phosphate reduced, **NAD⁺**: *β*-Nicotinamide adenine dinucleotide, **NADH**: *β*-Nicotinamide adenine dinucleotide reduced, **FAD**: Flavin adenine dinucleotide, **cAMP**: Cyclic adenosine monophosphate, **dUMP**: Deoxyuridine monophosphate, **dCMP**: Deoxycytidine monophosphate, **cGMP**: Cyclic guanosine monophosphate, **dGMP**: Deoxyguanosine monophosphate, **ADP**: Adenosine 5′-diphosphate, **GDP**: Guanosine 5′-diphosphate, **UDP**: Uridine 5′-diphosphate, **ATP**: Adenosine 5′-triphosphate, **CTP**: Cytidine 5′-triphosphate, **GTP**: Guanosine 5′-triphosphate, **TMP**: Thymidine 5′-monophosphate, **UMP**: Uridine 5′-monophosphate, **CMP**: Cytidine 5′-monophosphate, **GMP**: Guanosine 5′-monophosphate, **AMP**: Adenosine 5′-monophosphate, **XMP**: Xanthosine monophosphate, **IMP**: Inosine 5′-monophosphate, **RMP**: Riboflavin 5′-monophosphate, **DHAP**: Dihydroxyacetone phosphate, **KDPG**: 3-Deoxy-2-keto-6-phosphogluconic, **GA3P**: Glyceraldehyde 3-phosphate, **2-PGA/3-PGA**: 2-Phosphoglyceric acid/3-Phosphoglyceric acid, **E4P**: D-Erythrose 4-phosphate, **R5P**:

Ribose 5-phosphate, **Ru5P**: Ribulose-5-phosphate, **F1,6BP**: Fructose 1,6-bisphosphate, **R1,5BP**: Ribulose-1,5-bisphosphate, **G6P**: Glucose 6-phosphate, **F6P**: D-Fructose 6-phosphate, **S7P**: Sedoheptulose 7-phosphate.

Table **S2**. Retention time (RT), MRM transition (MT) and acquisition mode of ten isotope internal standards in HILIC-MS/MS method.

| No. | Category | Compound | RT(min) | MT | Mode |
|---|---|---|---|---|---|
| 1 | Amino Acid | Glutamine (U-$^{13}C_5$, U-$^{15}N_2$) | 6.85 | 154.1 > 89.1 | ES+ |
| 2 | Amino Acid | Isoleucine ($^{13}C_6$,$^{15}N$) | 5.55 | 139.2 > 92.0 | ES+ |
| 3 | Amino Acid | Alanine (2,3-$^{13}C_2$) | 6.49 | 92.1 > 46.1 | ES+ |
| 4 | Amino Acid | Aspartic acid (U-$^{13}$C4,$^{15}$N) | 7.69 | 139.2 > 92.1 | ES+ |
| 5 | Nucleotide | AMP ($^{13}C_{10}$,$^{15}N_5$) | 8.22 | 363.1 > 146.1 | ES+ |
| 6 | Organic acid | Malic acid ($^{13}C_4$) | 7.35 | 137.1 > 119.0 | ES- |
| 7 | Organic acid | Succinate (1,4-$^{13}C_2$) | 7.06 | 119.0 > 74.0 | ES- |
| 8 | Organic acid | Pyruvate ($^{13}$C) | 4.93 | 88.0 > 44.0 | ES- |
| 9 | Coenzyme | Palmitoyl-CoA ($^{13}C_{16}$) | 4.82 | 1022.5 > 515.5 | ES+ |
| 10 | Nucleobase | Uracil (1,3-$^{15}N_2$) | 3.02 | 113.0 > 43.0 | ES- |

Table **S3**. Details of LC gradients in optimization of HILIC condition.

| Gradient A | | | Gradient B | | | Gradient C | | |
|---|---|---|---|---|---|---|---|---|
| Time (min) | Flow (mL/min) | Solvent B (%) | Time (min) | Flow (mL/min) | Solvent B (%) | Time (min) | Flow (mL/min) | Solvent B (%) |
| 0.0 | 0.15 | 100 | 0.0 | 0.15 | 100 | 0.0 | 0.15 | 100 |
| 0.2 | 0.15 | 100 | 1.0 | 0.20 | 100 | 0.5 | 0.15 | 100 |
| 10.0 | 0.25 | 30 | 6.0 | 0.20 | 30 | 1.0 | 0.15 | 80 |
| 15.0 | 0.30 | 30 | 16.0 | 0.20 | 30 | 8.0 | 0.30 | 70 |
| 16.0 | 0.30 | 2 | 17.0 | 0.30 | 2 | 10.0 | 0.35 | 30 |
| 18.0 | 0.30 | 2 | 23.0 | 0.30 | 2 | 11.0 | 0.35 | 0 |
| 19.0 | 0.30 | 2 | 24.0 | 0.30 | 100 | 14.0 | 0.35 | 0 |
| 25.0 | 0.40 | 100 | 25.0 | 0.30 | 100 | 14.1 | 0.40 | 100 |
| 30.0 | 0.40 | 100 | 26.0 | 0.40 | 100 | 23.5 | 0.40 | 100 |
| | | | 30.0 | 0.40 | 100 | 23.7 | 0.15 | 100 |
| | | | | | | 25.0 | 0.15 | 100 |

Table **S4**. Stock and working standard Solutions for HILIC-UPLC-MS/MS method. Metabolites were classified in two different concentration groups (A and B) according to the responds. Stock solutions of analytes should be prepared in concentration of 20 μM for group A and 200 μM for group B in methanol (MeOH)/water, 1:1 (v/v), calibration standards (11 standards) should be prepared by serial dilution of the highest concentration standard.

| No. | Category | Compound | Group | No. | Category | Compound | Group |
|---|---|---|---|---|---|---|---|
| 1 | Amino Acid | 3-Guanidipropionic | B | 53 | Nucleobase | Uracil | B |
| 2 | Amino Acid | Phenylalanine | A | 54 | Nucleobase | Hypoxanthine | A |
| 3 | Amino Acid | Histidine | A | 55 | Nucleobase | Adenine | A |
| 4 | Amino Acid | Tryptophan | B | 56 | Nucleobase | Guanosine | A |
| 5 | Amino Acid | Isoleucine | A | 57 | Nucleobase | Cytidine | A |
| 6 | Amino Acid | leucine | A | 58 | Nucleobase | Cytosine | A |
| 7 | Amino Acid | Asparagine | A | 59 | Nucleobase | Creatinine | A |
| 8 | Amino Acid | Hydroxyproline | A | 60 | Nucleobase | Inosine | A |
| 9 | Amino Acid | Serine | A | 61 | Nucleobase | Guanine | A |
| 10 | Amino Acid | Glutamine | A | 62 | Nucleobase | Uridine | A |
| 11 | Amino Acid | Proline | A | 63 | Nucleobase | Xanthine | B |
| 12 | Amino Acid | Alanine | A | 64 | Nucleobase | Thymine | A |
| 13 | Amino Acid | Arginine | A | 65 | Organic Acid | Itaconic acid | B |
| 14 | Amino Acid | Threonine/Homoserine | A | 66 | Organic Acid | α-ketoglutaric acid | A |
| 15 | Amino Acid | Ornithine | A | 67 | Organic Acid | Fumarate | B |
| 16 | Amino Acid | Tyrosine | A | 68 | Organic Acid | Hippuric acid | B |
| 17 | Amino Acid | Lysine | A | 69 | Organic Acid | Malic acid | B |
| 18 | Amino Acid | Glycine | B | 70 | Organic Acid | Oritic acid | B |
| 19 | Amino Acid | Aspartic acid | A | 71 | Organic Acid | Lactate | B |
| 20 | Amino Acid | Methionine | A | 72 | Organic Acid | Aconitic acid | B |
| 21 | Amino Acid | Pyroglutamic acid | A | 73 | Organic Acid | Shikimic acid | B |
| 22 | Amino Acid | Theanine | A | 74 | Organic Acid | Succinate | B |

| | | | | | | | |
|---|---|---|---|---|---|---|---|
| 23 | Amino Acid | Valine | A | 75 | Organic Acid | Pyruvate | B |
| 24 | Amino Acid derivative | Taurine | A | 76 | Organic Acid | Glyceric acid | B |
| 25 | Amino Acid derivative | GSSG | A | 77 | Organic Acid | Citrate/Isocitrate | B |
| 26 | Amino Acid derivative | GSH | B | 78 | Phosphorylated compound | DHAP | B |
| 27 | Coenzyme | Acetyl CoA | A | 79 | Phosphorylated compound | Phosphocreatine | B |
| 28 | Coenzyme | $NADP^+$ | A | 80 | Phosphorylated compound | β-glyceric phosphate | B |
| 29 | Coenzyme | NADPH | B | 81 | Phosphorylated compound | α-glyceric phosphate | B |
| 30 | Coenzyme | $NAD^+$ | A | 82 | Phosphorylated compound | KDPG | B |
| 31 | Coenzyme | NADH | A | 83 | Phosphorylated compound | GA3P | B |
| 32 | Coenzyme | FAD | B | 84 | Phosphorylated compound | 2-PGA/3-PGA | B |
| 33 | Coenzyme | Palmitoyl CoA | A | 85 | Phosphorylated compound | Phosphoenolpyruvate | B |
| 34 | Nucleotide | cAMP | A | 86 | Phosphorylated compound | 6-Phosphogluconate | B |
| 35 | Nucleotide | dUMP | A | 87 | Phosphorylated compound | E4P | B |
| 36 | Nucleotide | dCMP | A | 88 | Phosphorylated sugar | R5P | B |
| 37 | Nucleotide | cGMP | A | 89 | Phosphorylated sugar | Ru5P | B |

| | | | | | | | |
|---|---|---|---|---|---|---|---|
| 38 | Nucleotide | dGMP | A | 90 | Phosphorylated sugar | F1,6BP | B |
| 39 | Nucleotide | ADP | A | 91 | Phosphorylated sugar | R1,5BP | B |
| 40 | Nucleotide | GDP | A | 92 | Phosphorylated sugar | G6P | B |
| 41 | Nucleotide | UDP | A | 93 | Phosphorylated sugar | F6P | B |
| 42 | Nucleotide | ATP | A | 94 | Phosphorylated sugar | S7P | B |
| 43 | Nucleotide | CTP | A | 95 | Sugar | Mannitol | B |
| 44 | Nucleotide | GTP | A | 96 | Sugar | Ribose | B |
| 45 | Nucleotide | TMP | A | 97 | Vitamin | Folic acid | B |
| 46 | Nucleotide | UMP | A | 98 | Vitamin | Pyridoxine | B |
| 47 | Nucleotide | CMP | A | 99 | Vitamin | Pantothenate | B |
| 48 | Nucleotide | GMP | A | 100 | Vitamin | Thiamine | A |
| 49 | Nucleotide | AMP | A | 101 | Vitamin | Riboflavin | A |
| 50 | Nucleotide | XMP | A | 102 | Vitamin | Choline | A |
| 51 | Nucleotide | IMP | A | 103 | Vitamin | Vit $B_{12}$ | A |
| 52 | Nucleotide | RMP | A | 104 | Vitamin | Niacinamide | A |

Table **S5**. Quantitation of polar metabolites in each sample treated by 3-BrPy with different concentration.

Value are expressed as nmol per 2.50 E+06 cells (N = 6).

| **No.** | **Class** | **compound** | **Quantitation (nmol)** | | | | | | | | | | | |
|---|---|---|---|---|---|---|---|---|---|---|---|---|---|---|
| | | | **Control** | | **5 µM 3-BrPy** | | **15 µM 3-BrPy** | | **50 µM 3-BrPy** | | **100 µM 3-BrPy** | | **Positive control** | |
| | | | **mean** | **SD** | **mean** | **SD** | **mean** | **SD** | **mean** | **SD** | **mean** | **SD** | **mean** | **SD** |
| 1 | Amino Acid | Arginine | 0.38 | 0.07 | 0.37 | 0.07 | 0.48 | 0.11 | 0.44 | 0.13 | 0.28 | 0.04 | 0.41 | 0.05 |
| 2 | Amino Acid | Asparagine | 0.14 | 0.02 | 0.13 | 0.01 | 0.14 | 0.02 | 0.12 | 0.01 | 0.11 | 0.03 | 0.21 | 0.02 |
| 3 | Amino Acid | Aspartic acid | 5.89 | 1.26 | 8.05 | 1.86 | 8.60 | 1.67 | 5.47 | 0.98 | 4.99 | 0.41 | 11.89 | 2.04 |
| 4 | Amino Acid | Alanine | 0.47 | 0.06 | 0.46 | 0.08 | 0.43 | 0.10 | 0.49 | 0.09 | 0.30 | 0.06 | 0.34 | 0.13 |
| 5 | Amino Acid | Glutamine | 13.10 | 3.07 | 12.72 | 1.96 | 10.57 | 2.08 | 8.38 | 1.89 | 7.01 | 1.08 | 8.39 | 1.94 |
| 6 | Amino Acid | Leucine | 0.03 | 0.02 | 0.04 | 0.02 | 0.04 | 0.02 | 0.09 | 0.01 | 0.08 | 0.01 | 0.13 | 0.03 |
| 7 | Amino Acid | Isoleucine | 1.98 | 0.29 | 2.02 | 0.36 | 2.90 | 0.68 | 2.95 | 0.62 | 2.08 | 0.22 | 3.44 | 0.42 |
| 8 | Amino Acid | Histidine | 2.38 | 0.42 | 1.96 | 0.28 | 2.62 | 0.31 | 2.51 | 0.82 | 2.42 | 0.16 | 2.50 | 0.51 |
| 9 | Amino Acid | Phenylalanine | 2.50 | 0.40 | 2.23 | 0.28 | 3.17 | 0.52 | 2.90 | 0.82 | 2.38 | 0.26 | 2.93 | 0.32 |
| 10 | Amino Acid | Methionine | 10.22 | 1.55 | 10.22 | 1.61 | 12.54 | 3.00 | 13.42 | 2.32 | 9.62 | 1.70 | 14.40 | 2.25 |
| 11 | Amino Acid | Hydroxyproline | 0.10 | 0.03 | 0.11 | 0.06 | 0.11 | 0.03 | 0.09 | 0.04 | 0.04 | 0.01 | 0.12 | 0.03 |
| 12 | Amino Acid | Proline | 0.12 | 0.02 | 0.10 | 0.02 | 0.14 | 0.04 | 0.10 | 0.03 | 0.05 | 0.02 | 0.10 | 0.04 |
| 13 | Amino Acid | Serine | 7.68 | 2.22 | 7.22 | 0.60 | 7.01 | 0.65 | 6.27 | 1.24 | 5.81 | 0.33 | 6.62 | 0.96 |
| 14 | Amino Acid | Lysine | 2.22 | 0.50 | 2.14 | 0.43 | 2.86 | 0.72 | 2.47 | 0.71 | 1.68 | 0.23 | 2.39 | 0.27 |
| 15 | Amino Acid | Ornithine | 0.07 | 0.02 | 0.06 | 0.01 | 0.08 | 0.01 | 0.05 | 0.01 | 0.05 | 0.01 | 0.06 | 0.02 |
| 16 | Amino Acid | Threonine | 0.94 | 0.22 | 0.87 | 0.27 | 1.35 | 0.39 | 1.38 | 0.14 | 0.79 | 0.08 | 1.47 | 0.28 |
| 17 | Amino Acid | Tryptophan | 7.24 | 1.65 | 7.99 | 1.62 | 10.50 | 2.67 | 9.15 | 2.51 | 10.34 | 1.13 | 10.35 | 2.35 |
| 18 | Amino Acid | Tyrosine | 3.63 | 1.62 | 3.50 | 1.29 | 4.29 | 1.65 | 5.59 | 1.97 | 4.80 | 0.79 | 4.37 | 1.38 |
| 19 | Amino Acid | Pyroglutamic acid | 4.40 | 0.88 | 3.75 | 0.43 | 4.20 | 0.45 | 3.01 | 0.65 | 2.38 | 0.21 | 2.98 | 0.63 |
| 20 | Amino Acid | Valine | 0.60 | 0.11 | 0.60 | 0.13 | 0.79 | 0.11 | 0.80 | 0.21 | 0.56 | 0.09 | 0.87 | 0.17 |
| 21 | Amino Acid derivative | GSH | 163.37 | 27.20 | 175.19 | 33.04 | 202.72 | 26.16 | 134.51 | 31.82 | 129.87 | 15.88 | 73.09 | 15.42 |
| 22 | Amino Acid derivative | GSSG | 1.15 | 0.15 | 0.98 | 0.26 | 0.93 | 0.21 | 1.07 | 0.14 | 0.94 | 0.08 | 1.01 | 0.10 |
| 23 | Amino Acid derivative | Taurine | 6.34 | 1.09 | 6.90 | 1.88 | 9.01 | 2.38 | 6.53 | 1.31 | 3.60 | 1.73 | 10.65 | 2.39 |
| 24 | Coenzyme | $NAD^+$ | 0.67 | 0.08 | 0.75 | 0.11 | 0.92 | 0.19 | 1.02 | 0.27 | 0.53 | 0.04 | 0.84 | 0.10 |
| 25 | Coenzyme | NADH | 2.07 | 0.54 | 1.72 | 0.36 | 1.50 | 0.37 | 1.12 | 0.51 | 1.08 | 0.21 | 0.28 | 0.06 |
| 26 | Coenzyme | NADP | 0.02 | 0.01 | 0.01 | 0.00 | 0.01 | 0.01 | 0.01 | 0.00 | 0.01 | 0.00 | 0.01 | 0.00 |
| 27 | Coenzyme | FAD | 0.13 | 0.05 | 0.14 | 0.09 | 0.15 | 0.05 | 0.10 | 0.02 | 0.11 | 0.04 | 0.10 | 0.02 |
| 28 | Coenzyme | Acetyl CoA | 0.07 | 0.01 | 0.07 | 0.02 | 0.07 | 0.03 | 0.04 | 0.02 | 0.01 | 0.00 | 0.08 | 0.01 |
| 29 | Coenzyme | Pamitoyl-CoA | 0.02 | 0.00 | 0.02 | 0.00 | 0.01 | 0.00 | 0.01 | 0.01 | 0.00 | 0.00 | 0.01 | 0.01 |

| | | | | | | | | | | | | | | |
|---|---|---|---|---|---|---|---|---|---|---|---|---|---|---|
| 30 | Nucleobase | Adenine | 0.01 | 0.00 | 0.01 | 0.00 | 0.02 | 0.01 | 0.02 | 0.00 | 0.02 | 0.00 | 0.02 | 0.00 |
| 31 | Nucleobase | Cytidine | 0.01 | 0.01 | 0.02 | 0.01 | 0.02 | 0.01 | 0.02 | 0.01 | 0.03 | 0.01 | 0.01 | 0.00 |
| 32 | Nucleobase | Creatinine | 0.06 | 0.01 | 0.06 | 0.02 | 0.08 | 0.01 | 0.06 | 0.02 | 0.03 | 0.02 | 0.06 | 0.01 |
| 33 | Nucleobase | Guanine | 0.11 | 0.06 | 0.09 | 0.03 | 0.11 | 0.07 | 0.16 | 0.08 | 0.21 | 0.15 | 0.05 | 0.03 |
| 34 | Nucleobase | Guanosine | 0.06 | 0.03 | 0.06 | 0.02 | 0.06 | 0.03 | 0.09 | 0.04 | 0.12 | 0.06 | 0.04 | 0.03 |
| 35 | Nucleobase | Inosine | 0.03 | 0.01 | 0.04 | 0.01 | 0.03 | 0.01 | 0.03 | 0.01 | 0.03 | 0.01 | 0.03 | 0.00 |
| 36 | Nucleobase | Hypoxanthine | 0.19 | 0.04 | 0.19 | 0.04 | 0.21 | 0.02 | 0.19 | 0.04 | 0.17 | 0.03 | 0.21 | 0.02 |
| 37 | Nucleobase | Thymine | 0.12 | 0.01 | 0.13 | 0.01 | 0.17 | 0.08 | 0.14 | 0.01 | 0.15 | 0.03 | 0.16 | 0.01 |
| 38 | Nucleobase | Uracil | 0.12 | 0.01 | 0.14 | 0.05 | 0.13 | 0.01 | 0.15 | 0.02 | 0.12 | 0.01 | 0.14 | 0.01 |
| 39 | Nucleobase | Uridine | 0.22 | 0.02 | 0.24 | 0.03 | 0.25 | 0.06 | 0.27 | 0.03 | 0.31 | 0.08 | 0.24 | 0.02 |
| 40 | Nucleotide | ADP | 2.37 | 0.52 | 2.43 | 0.27 | 2.60 | 0.33 | 2.44 | 0.49 | 2.53 | 0.49 | 1.43 | 0.19 |
| 41 | Nucleotide | UDP | 0.41 | 0.15 | 0.37 | 0.08 | 0.36 | 0.11 | 0.39 | 0.12 | 0.46 | 0.10 | 0.10 | 0.02 |
| 42 | Nucleotide | GDP | 0.67 | 0.13 | 0.67 | 0.07 | 0.74 | 0.07 | 0.65 | 0.11 | 0.66 | 0.09 | 0.44 | 0.06 |
| 43 | Nucleotide | ATP | 9.28 | 0.75 | 8.11 | 1.41 | 8.26 | 1.42 | 6.59 | 0.97 | 6.40 | 0.39 | 6.68 | 0.77 |
| 44 | Nucleotide | GTP | 1.80 | 0.35 | 1.73 | 0.49 | 1.64 | 0.39 | 1.21 | 0.32 | 0.98 | 0.15 | 1.01 | 0.23 |
| 45 | Nucleotide | CTP | 1.90 | 0.60 | 1.82 | 0.45 | 1.70 | 0.27 | 1.35 | 0.20 | 1.22 | 0.16 | 0.96 | 0.22 |
| 46 | Nucleotide | dCMP | 0.01 | 0.00 | 0.02 | 0.01 | 0.02 | 0.01 | 0.02 | 0.00 | 0.01 | 0.00 | 0.02 | 0.00 |
| 47 | Nucleotide | IMP | 0.03 | 0.01 | 0.03 | 0.01 | 0.03 | 0.01 | 0.02 | 0.00 | 0.02 | 0.01 | 0.02 | 0.00 |
| 48 | Nucleotide | AMP | 0.59 | 0.15 | 0.62 | 0.21 | 0.97 | 0.24 | 1.02 | 0.33 | 0.47 | 0.12 | 0.29 | 0.06 |
| 49 | Nucleotide | CMP | 0.05 | 0.01 | 0.05 | 0.01 | 0.05 | 0.00 | 0.05 | 0.00 | 0.05 | 0.01 | 0.06 | 0.00 |
| 50 | Nucleotide | UMP | 0.09 | 0.02 | 0.09 | 0.02 | 0.08 | 0.02 | 0.07 | 0.01 | 0.03 | 0.00 | 0.04 | 0.01 |
| 51 | Nucleotide | XMP | 0.10 | 0.03 | 0.13 | 0.04 | 0.20 | 0.06 | 0.16 | 0.04 | 0.08 | 0.02 | 0.13 | 0.03 |
| 52 | Organic acid | Pyruvate | 0.80 | 0.39 | 0.98 | 0.76 | 0.57 | 0.33 | 0.68 | 0.38 | 1.29 | 0.18 | 1.09 | 0.52 |
| 53 | Organic acid | Fumarate | 1.34 | 0.14 | 2.09 | 0.50 | 1.87 | 0.53 | 2.51 | 0.50 | 0.98 | 1.00 | 0.51 | 0.12 |
| 54 | Organic acid | Succinate | 1.48 | 0.32 | 1.50 | 0.31 | 1.42 | 0.21 | 1.50 | 0.19 | 1.10 | 0.23 | 1.11 | 0.24 |
| 55 | Organic acid | Aconitic acid | 0.66 | 0.10 | 0.75 | 0.13 | 0.68 | 0.15 | 0.84 | 0.18 | 1.07 | 0.17 | 0.46 | 0.05 |
| 56 | Organic acid | Citrate (Isocitrate) | 5.59 | 1.02 | 6.17 | 0.45 | 5.59 | 0.44 | 5.43 | 1.54 | 5.85 | 0.50 | 4.18 | 0.55 |
| 57 | Organic acid | Malic acid | 3.93 | 0.83 | 4.54 | 0.74 | 4.02 | 0.61 | 4.23 | 0.77 | 3.26 | 0.75 | 0.91 | 0.28 |
| 58 | Organic acid | Shikimic acid | 0.12 | 0.00 | 0.12 | 0.00 | 0.13 | 0.00 | 0.13 | 0.00 | 0.13 | 0.00 | 0.15 | 0.01 |
| 59 | Organic acid | Lactate | 9.84 | 1.54 | 10.97 | 1.58 | 18.57 | 4.94 | 26.74 | 7.72 | 14.60 | 4.43 | 3.00 | 0.90 |
| 60 | Organic acid | α-ketoglutaric acid | 0.07 | 0.03 | 0.12 | 0.05 | 0.16 | 0.08 | 0.12 | 0.08 | 0.06 | 0.03 | 0.09 | 0.04 |
| 61 | Phosphorylated compound | 2-PGA (3-PGA) | 24.43 | 6.25 | 21.41 | 5.33 | 19.40 | 2.70 | 15.04 | 4.60 | 16.88 | 2.40 | 17.98 | 1.93 |
| 62 | Phosphorylated compound | α-glycerol phosphate | 0.09 | 0.01 | 0.12 | 0.05 | 0.11 | 0.02 | 0.11 | 0.02 | 0.09 | 0.01 | 0.12 | 0.01 |
| 63 | Phosphorylated compound | GA3P | 0.25 | 0.12 | 0.23 | 0.08 | 0.26 | 0.12 | 0.26 | 0.03 | 0.17 | 0.07 | 0.06 | 0.05 |
| 64 | Phosphorylated compound | RMP | 0.03 | 0.00 | 0.03 | 0.00 | 0.03 | 0.00 | 0.02 | 0.00 | 0.02 | 0.00 | 0.03 | 0.01 |
| 65 | Phosphorylated compound | Phosphoenolpyruvate | 0.03 | 0.02 | 0.02 | 0.01 | 0.03 | 0.01 | 0.02 | 0.01 | 0.01 | 0.01 | 0.03 | 0.01 |

| | | | | | | | | | | | | | | |
|---|---|---|---|---|---|---|---|---|---|---|---|---|---|---|
| 66 | Phosphorylated sugar | G6P | 1.93 | 0.74 | 1.97 | 0.40 | 3.30 | 1.10 | 2.60 | 0.78 | 2.11 | 1.78 | 0.76 | 0.08 |
| 67 | Phosphorylated sugar | R5P | 0.35 | 0.09 | 0.37 | 0.18 | 0.40 | 0.18 | 0.41 | 0.11 | 0.45 | 0.10 | 0.25 | 0.01 |
| 68 | Phosphorylated sugar | S7P | 0.28 | 0.03 | 0.28 | 0.03 | 0.26 | 0.05 | 0.27 | 0.04 | 0.24 | 0.06 | 0.22 | 0.02 |
| 69 | Phosphorylated sugar | F1,6BP | 5.70 | 1.82 | 4.46 | 0.94 | 4.45 | 1.08 | 2.66 | 0.37 | 2.21 | 0.68 | 0.19 | 0.04 |
| 70 | Phosphorylated sugar | F6P | 0.99 | 0.22 | 0.91 | 0.13 | 0.99 | 0.13 | 0.82 | 0.09 | 0.60 | 0.09 | 0.50 | 0.01 |
| 71 | Sugar | Mannitol | 0.08 | 0.04 | 0.14 | 0.08 | 0.31 | 0.27 | 0.35 | 0.25 | 0.05 | 0.05 | 0.50 | 0.49 |
| 72 | Vitamin | Niacinamide | 0.03 | 0.02 | 0.04 | 0.01 | 0.09 | 0.04 | 0.06 | 0.02 | 0.12 | 0.08 | 0.06 | 0.01 |
| 73 | Vitamin | Pyridoxine | 1.17 | 0.24 | 1.32 | 0.27 | 2.08 | 0.23 | 1.59 | 0.45 | 0.78 | 0.63 | 1.62 | 0.25 |
| 74 | Vitamin | Riboflavin | 0.00 | 0.00 | 0.00 | 0.00 | 0.00 | 0.00 | 0.00 | 0.00 | 0.00 | 0.00 | 0.00 | 0.00 |
| 75 | Vitamin | Pantothenate | 7.03 | 5.38 | 5.66 | 1.85 | 6.18 | 1.20 | 7.25 | 2.64 | 5.00 | 6.24 | 3.26 | 0.80 |
| 76 | Vitamin | Choline | 0.61 | 0.14 | 0.83 | 0.09 | 0.92 | 0.06 | 0.95 | 0.17 | 0.76 | 0.05 | 1.00 | 0.16 |
| 77 | Vitamin | Folic acid | 0.62 | 0.13 | 0.74 | 0.04 | 0.88 | 0.21 | 0.89 | 0.15 | 0.69 | 0.07 | 1.11 | 0.13 |
| 78 | Vitamin | Thiamine | 0.04 | 0.02 | 0.05 | 0.02 | 0.03 | 0.01 | 0.06 | 0.03 | 0.01 | 0.01 | 0.07 | 0.02 |

Table **S6**. Quantitation of polar metabolites in each sample treated by TCDD with different concentration with 4 hours and 24 hours. Value are expressed as pmol, the cell number for each sample was approximately 1.0 E+06 (N = 6).

| **No.** | **Class** | **Compound** | **Quantitation (pmol)** | | | | | |
|---|---|---|---|---|---|---|---|---|
| | | | **DMSO_4 h** | | **1 nM_4 h** | | **10 nM_4 h** | |
| | | | **mean** | **SD** | **mean** | **SD** | **mean** | **SD** |
| 1 | Amino Acid | Alanine | 26.07 | 26.91 | 11.71 | 6.91 | 19.68 | 12.92 |
| 2 | Amino Acid | Serine | 375.65 | 260.77 | 462.74 | 270.83 | 702.17 | 391.30 |
| 3 | Amino Acid | Proline | 612.11 | 207.35 | 704.30 | 337.39 | 532.89 | 172.38 |
| 4 | Amino Acid | Valine | 472.75 | 210.38 | 618.94 | 114.64 | 564.99 | 329.35 |
| 5 | Amino Acid | Threonine | 375.59 | 81.65 | 492.65 | 80.23 | 714.23 | 215.94 |
| 6 | Amino Acid | Pyroglutamic acid | 16170.69 | 4318.92 | 15255.17 | 815.24 | 21301.88 | 4343.70 |
| 7 | Amino Acid | Isoleucine | 1234.91 | 663.12 | 1753.07 | 588.86 | 2941.08 | 1549.52 |
| 8 | Amino Acid | Hydroxyproline | 177.33 | 77.12 | 168.35 | 82.64 | 155.88 | 40.26 |
| 9 | Amino Acid | Asparagine | 204.83 | 75.02 | 209.20 | 76.85 | 295.30 | 84.42 |
| 10 | Amino Acid | Aspartic acid | 2598.65 | 726.55 | 2824.75 | 747.84 | 3706.93 | 698.46 |
| 11 | Amino Acid | Glutamine | 13133.63 | 3935.16 | 12084.56 | 881.14 | 17350.44 | 3340.89 |
| 12 | Amino Acid | Lysine | 531.80 | 231.61 | 902.91 | 323.50 | 1461.52 | 1084.24 |
| 13 | Amino Acid | Methionine | 17885.33 | 3405.82 | 25928.75 | 7334.92 | 36297.18 | 13069.66 |
| 14 | Amino Acid | Histidine | 1256.55 | 631.77 | 1419.50 | 625.80 | 2748.08 | 1296.25 |
| 15 | Amino Acid | Phenylalanine | 720.46 | 143.10 | 1097.83 | 252.14 | 1525.21 | 686.42 |

| | | | | | | | | |
|---|---|---|---|---|---|---|---|---|
| 16 | Amino Acid | Arginine | 55.49 | 19.87 | 98.65 | 18.39 | 194.36 | 179.66 |
| 17 | Amino Acid | Tyrosine | 1301.39 | 243.87 | 1896.74 | 406.31 | 3227.50 | 1889.43 |
| 18 | Amino Acid | Tryptophan | 412.44 | 143.91 | 597.69 | 181.06 | 711.26 | 438.24 |
| 19 | Amino Acid derivative | Taurine | 5340.18 | 1137.09 | 6258.98 | 2130.57 | 5492.20 | 1243.31 |
| 20 | Amino Acid derivative | GSSG | 4041.01 | 1359.41 | 3660.92 | 1114.88 | 5390.80 | 1391.51 |
| 21 | Amino Acid derivative | GSH | 543945.28 | 243820.86 | 590689.82 | 160092.84 | 579492.81 | 153398.04 |
| 22 | Nucleobase | Adenine | 11.21 | 3.30 | 8.86 | 1.72 | 10.51 | 3.02 |
| 23 | Nucleobase | Creatinine | 50.15 | 24.56 | 48.52 | 11.55 | 61.14 | 24.20 |
| 24 | Nucleobase | Xanthine | 18.08 | 6.98 | 18.74 | 10.02 | 21.68 | 5.94 |
| 25 | Nucleotide | Cytidine | 8.79 | 1.94 | 7.21 | 0.57 | 9.93 | 3.97 |
| 26 | Nucleotide | Guanosine | 18.72 | 1.18 | 19.08 | 1.89 | 18.74 | 1.08 |
| 27 | Nucleotide | Uracil | 26.11 | 20.69 | 13.76 | 4.99 | 11.45 | 5.36 |
| 28 | Nucleotide | GMP | 296.85 | 4.83 | 296.53 | 5.77 | 299.67 | 7.89 |
| 29 | Nucleotide | CMP | 39.68 | 3.67 | 40.02 | 6.99 | 37.28 | 2.85 |
| 30 | Nucleotide | UMP | 293.33 | 78.30 | 300.60 | 102.17 | 271.64 | 54.14 |
| 31 | Nucleotide | AMP | 835.41 | 566.04 | 1007.10 | 869.68 | 1967.63 | 1625.36 |
| 32 | Nucleotide | IMP | 33.75 | 13.48 | 27.94 | 8.14 | 30.82 | 12.18 |
| 33 | Nucleotide | RMP | 110.55 | 31.65 | 115.15 | 27.51 | 148.49 | 29.98 |
| 34 | Nucleotide | XMP | 8.21 | 1.97 | 9.63 | 3.35 | 8.07 | 2.58 |
| 35 | Nucleotide | dUMP | 260.14 | 160.97 | 317.07 | 131.86 | 269.83 | 75.99 |

| | | | | | | | | |
|---|---|---|---|---|---|---|---|---|
| 36 | Nucleotide | cAMP | 2.32 | 0.40 | 2.26 | 0.42 | 2.33 | 0.32 |
| 37 | Nucleotide | dGMP | 26.67 | 1.30 | 25.37 | 1.69 | 25.62 | 2.89 |
| 38 | Nucleotide | UDP | 3030.89 | 1383.71 | 3509.01 | 1695.00 | 5838.16 | 3160.42 |
| 39 | Nucleotide | ADP | 7390.50 | 3143.66 | 8635.94 | 4493.89 | 15114.87 | 9551.01 |
| 40 | Nucleotide | GDP | 1134.31 | 349.30 | 1152.67 | 305.02 | 1196.06 | 191.78 |
| 41 | Nucleotide | CTP | 7474.37 | 3468.33 | 7655.18 | 2680.49 | 14485.88 | 7937.00 |
| 42 | Nucleotide | ATP | 25693.21 | 10500.79 | 28480.17 | 11390.20 | 47650.92 | 24081.01 |
| 43 | Nucleotide | GTP | 9360.11 | 4597.01 | 10773.01 | 4694.61 | 19224.17 | 10436.86 |
| 44 | Coenzyme | $NAD^+$ | 868.48 | 169.53 | 878.19 | 197.07 | 1073.25 | 69.62 |
| 45 | Coenzyme | $NADP^+$ | 15.67 | 8.92 | 16.35 | 10.71 | 16.80 | 7.32 |
| 46 | Coenzyme | FAD | 95.17 | 12.66 | 98.79 | 19.46 | 105.19 | 3.80 |
| 47 | Coenzyme | NADH | 6436.31 | 4451.45 | 8028.38 | 3468.92 | 11191.08 | 3031.42 |
| 48 | Coenzyme | Acetyl-CoA | 71.36 | 15.30 | 76.78 | 11.06 | 81.37 | 12.09 |
| 49 | Coenzyme | Palmitoyl-CoA | 23.37 | 3.00 | 22.49 | 2.09 | 22.76 | 0.81 |
| 50 | Coenzyme | Stearoyl-CoA | 8.66 | 1.37 | 11.25 | 4.41 | 9.81 | 2.63 |
| 51 | Organic Acid | Lactate | 3696.71 | 2189.71 | 6744.70 | 966.56 | 8730.32 | 2629.83 |
| 52 | Organic Acid | Succinate | 282.91 | 65.38 | 392.74 | 183.83 | 438.98 | 95.78 |
| 53 | Organic Acid | Malic acid | 3985.61 | 1032.22 | 5175.18 | 1976.22 | 8108.87 | 2315.05 |
| 54 | Organic Acid | Hippuric acid | 204.59 | 11.30 | 200.81 | 3.91 | 202.62 | 5.74 |
| 55 | Organic Acid | Itaconic acid | 221.36 | 176.99 | 272.91 | 279.71 | 406.51 | 143.08 |

| | | | | | | | | |
|---|---|---|---|---|---|---|---|---|
| 56 | Organic Acid | Aconitic acid | 19.56 | 15.91 | 16.27 | 6.25 | 34.41 | 11.84 |
| 57 | Organic Acid | Citric acid | 1431.29 | 76.68 | 1395.86 | 38.93 | 1501.05 | 63.30 |
| 58 | Sugar | Ribose | 8038.43 | 2156.39 | 13590.40 | 4558.65 | 17072.86 | 7096.13 |
| 59 | Sugar | Mannitol | 52.07 | 12.43 | 70.25 | 25.13 | 83.18 | 39.67 |
| 60 | Phosphorylated sugar | Ru5P (R5P) | 1861.86 | 5.04 | 1867.98 | 6.77 | 1861.31 | 3.64 |
| 61 | Phosphorylated sugar | F6P | 620.81 | 49.67 | 682.41 | 102.05 | 641.81 | 34.53 |
| 62 | Phosphorylated sugar | G6P | 202.55 | 46.48 | 239.11 | 30.04 | 312.87 | 90.52 |
| 63 | Phosphorylated sugar | S7P | 387.41 | 68.44 | 482.45 | 33.70 | 461.91 | 70.33 |
| 64 | Phosphorylated sugar | F1,6BP | 2899.79 | 796.95 | 2968.42 | 785.98 | 3825.52 | 923.75 |
| 65 | Phosphorylated compound | GA3P | 100.29 | 45.41 | 133.98 | 82.86 | 182.17 | 85.41 |
| 66 | Phosphorylated compound | *α*-glyceric phosphate | 658.42 | 175.38 | 778.27 | 210.22 | 817.57 | 296.43 |
| 67 | Phosphorylated compound | 2-PGA (3-PGA) | 126481.82 | 18891.01 | 129873.47 | 12180.42 | 127414.91 | 18856.31 |
| 68 | Phosphorylated compound | Phosphoenolpyruvate | 370.84 | 6.22 | 381.07 | 20.31 | 398.05 | 38.99 |
| 69 | Vitamin | Pyridoxine | 23.40 | 7.84 | 26.77 | 7.73 | 46.55 | 20.59 |
| 70 | Vitamin | Riboflavin | 11.05 | 1.35 | 12.44 | 1.08 | 13.74 | 2.51 |
| 71 | Vitamin | Pantothenate | 1768.26 | 678.92 | 1543.98 | 403.15 | 2032.49 | 429.18 |
| 72 | Vitamin | Niacinamide | 14.33 | 4.02 | 19.98 | 8.14 | 31.58 | 20.48 |
| 73 | Vitamin | Choline | 380.67 | 137.99 | 358.39 | 199.87 | 395.35 | 65.96 |
| 74 | Vitamin | Thiamine | 127.56 | 42.39 | 199.54 | 96.43 | 184.84 | 33.40 |
| **No.** | **Class** | **Compound** | **DMSO_24 h** | | **1 nM_24 h** | | **10 nM_24 h** | |

| | | | mean | SD | mean | SD | mean | SD |
|---|---|---|---|---|---|---|---|---|
| 1 | Amino Acid | Alanine | 64.83 | 27.57 | 38.35 | 25.30 | 28.28 | 17.97 |
| 2 | Amino Acid | Serine | 2186.73 | 830.97 | 1102.20 | 531.24 | 617.87 | 428.15 |
| 3 | Amino Acid | Proline | 1072.58 | 369.43 | 667.87 | 123.69 | 731.52 | 201.34 |
| 4 | Amino Acid | Valine | 695.96 | 206.16 | 609.41 | 148.80 | 579.67 | 285.08 |
| 5 | Amino Acid | Threonine | 1001.58 | 159.08 | 707.31 | 195.39 | 604.42 | 91.40 |
| 6 | Amino Acid | Pyroglutamic acid | 32191.68 | 3225.50 | 23642.65 | 5885.62 | 15203.48 | 2853.11 |
| 7 | Amino Acid | Isoleucine | 3844.80 | 576.37 | 2481.98 | 501.15 | 2074.63 | 487.98 |
| 8 | Amino Acid | Hydroxyproline | 437.74 | 99.17 | 183.75 | 21.49 | 208.94 | 82.35 |
| 9 | Amino Acid | Asparagine | 1284.38 | 514.66 | 387.32 | 300.22 | 241.62 | 124.23 |
| 10 | Amino Acid | Aspartic acid | 4665.38 | 575.76 | 3238.41 | 802.43 | 2763.35 | 843.10 |
| 11 | Amino Acid | Glutamine | 28760.63 | 3379.73 | 20914.67 | 5565.04 | 12669.03 | 1701.51 |
| 12 | Amino Acid | Lysine | 1604.00 | 660.01 | 942.85 | 300.52 | 872.51 | 363.52 |
| 13 | Amino Acid | Methionine | 52180.95 | 6607.05 | 32535.46 | 3863.59 | 27067.46 | 2923.63 |
| 14 | Amino Acid | Histidine | 4179.44 | 1112.61 | 2952.17 | 866.80 | 1782.87 | 364.43 |
| 15 | Amino Acid | Phenylalanine | 1891.04 | 380.19 | 1215.32 | 195.39 | 1150.78 | 226.85 |
| 16 | Amino Acid | Arginine | 27.07 | 13.84 | 42.20 | 19.41 | 56.88 | 16.64 |
| 17 | Amino Acid | Tyrosine | 2614.35 | 450.13 | 2092.72 | 559.17 | 2046.17 | 912.46 |
| 18 | Amino Acid | Tryptophan | 822.86 | 124.22 | 553.97 | 170.68 | 484.38 | 217.22 |
| 19 | Amino Acid derivative | Taurine | 7277.23 | 2229.74 | 5607.35 | 926.15 | 7062.56 | 1618.63 |

| | | | | | | | | |
|---|---|---|---|---|---|---|---|---|
| 20 | Amino Acid derivative | GSSG | 5819.16 | 2239.13 | 5126.11 | 774.57 | 5238.39 | 1147.79 |
| 21 | Amino Acid derivative | GSH | 659128.64 | 292416.10 | 783888.54 | 170497.73 | 785053.50 | 108184.15 |
| 22 | Nucleobase | Adenine | 13.25 | 4.30 | 10.07 | 2.88 | 9.82 | 1.82 |
| 23 | Nucleobase | Creatinine | 91.38 | 23.19 | 71.98 | 6.86 | 64.66 | 10.92 |
| 24 | Nucleobase | Xanthine | 16.34 | 3.52 | 19.71 | 6.84 | 18.99 | 3.62 |
| 25 | Nucleotide | Cytidine | 9.96 | 1.17 | 8.39 | 1.29 | 7.97 | 0.98 |
| 26 | Nucleotide | Guanosine | 23.57 | 4.00 | 19.90 | 1.66 | 18.20 | 1.45 |
| 27 | Nucleotide | Uracil | 8.67 | 1.89 | 8.42 | 2.24 | 8.14 | 2.03 |
| 28 | Nucleotide | GMP | 303.99 | 8.66 | 299.10 | 4.56 | 301.90 | 9.51 |
| 29 | Nucleotide | CMP | 48.40 | 8.31 | 37.08 | 3.47 | 40.04 | 4.04 |
| 30 | Nucleotide | UMP | 431.70 | 104.49 | 302.47 | 61.56 | 272.74 | 62.60 |
| 31 | Nucleotide | AMP | 1577.36 | 906.52 | 1402.06 | 786.63 | 1161.38 | 579.89 |
| 32 | Nucleotide | IMP | 22.08 | 8.55 | 18.07 | 6.52 | 33.21 | 12.02 |
| 33 | Nucleotide | RMP | 202.03 | 20.31 | 178.81 | 34.28 | 159.84 | 42.24 |
| 34 | Nucleotide | XMP | 14.24 | 3.29 | 8.83 | 2.90 | 10.31 | 3.65 |
| 35 | Nucleotide | dUMP | 215.91 | 85.39 | 282.59 | 50.84 | 373.40 | 105.30 |
| 36 | Nucleotide | cAMP | 3.12 | 0.62 | 2.44 | 0.28 | 2.42 | 0.38 |
| 37 | Nucleotide | dGMP | 28.39 | 3.76 | 27.24 | 2.02 | 24.26 | 1.43 |
| 38 | Nucleotide | UDP | 6682.12 | 1616.66 | 5902.14 | 2830.01 | 5905.20 | 1818.07 |
| 39 | Nucleotide | ADP | 19225.68 | 8319.67 | 16109.67 | 7162.01 | 12872.87 | 3952.15 |

| | | | | | | | | |
|---|---|---|---|---|---|---|---|---|
| 40 | Nucleotide | GDP | 2549.80 | 408.61 | 1524.88 | 457.49 | 1769.35 | 218.94 |
| 41 | Nucleotide | CTP | 21831.31 | 8971.33 | 16536.23 | 7621.93 | 15611.21 | 4883.92 |
| 42 | Nucleotide | ATP | 91296.00 | 30135.80 | 63812.10 | 26947.34 | 53247.40 | 8753.39 |
| 43 | Nucleotide | GTP | 30756.41 | 7847.43 | 23177.71 | 9978.37 | 21329.36 | 5490.31 |
| 44 | Coenzyme | $NAD^+$ | 1975.15 | 328.29 | 1348.66 | 126.01 | 1310.83 | 151.41 |
| 45 | Coenzyme | $NADP^+$ | 37.88 | 15.96 | 22.59 | 11.92 | 28.32 | 8.67 |
| 46 | Coenzyme | FAD | 166.72 | 24.65 | 120.32 | 20.11 | 131.88 | 13.13 |
| 47 | Coenzyme | NADH | 27860.83 | 5775.18 | 21057.99 | 6072.07 | 17528.72 | 6132.44 |
| 48 | Coenzyme | Acetyl-CoA | 118.05 | 26.31 | 93.14 | 11.28 | 94.20 | 14.34 |
| 49 | Coenzyme | Palmitoyl-CoA | 35.58 | 6.19 | 31.16 | 7.71 | 27.16 | 2.78 |
| 50 | Coenzyme | Stearoyl-CoA | 19.20 | 2.34 | 14.30 | 2.27 | 10.55 | 2.44 |
| 51 | Organic Acid | Lactate | 12622.26 | 2967.07 | 10801.42 | 3510.44 | 14600.93 | 4786.93 |
| 52 | Organic Acid | Succinate | 504.05 | 141.16 | 491.98 | 135.51 | 538.75 | 105.28 |
| 53 | Organic Acid | Malic acid | 9724.17 | 1091.37 | 9727.80 | 2610.34 | 10513.18 | 2457.49 |
| 54 | Organic Acid | Hippuric acid | 200.39 | 1.47 | 201.36 | 3.89 | 199.29 | 1.26 |
| 55 | Organic Acid | Itaconic acid | 530.48 | 242.51 | 460.33 | 112.61 | 292.73 | 185.92 |
| 56 | Organic Acid | Aconitic acid | 82.35 | 28.52 | 50.72 | 10.39 | 37.11 | 4.42 |
| 57 | Organic Acid | Citric acid | 1695.51 | 79.15 | 1567.62 | 65.23 | 1507.82 | 31.92 |
| 58 | Sugar | Ribose | 26514.72 | 5192.75 | 22123.98 | 8614.85 | 18295.21 | 5042.65 |
| 59 | Sugar | Mannitol | 48.55 | 9.87 | 48.99 | 6.19 | 56.40 | 11.34 |

| | | | | | | | | |
|---|---|---|---|---|---|---|---|---|
| 60 | Phosphorylated sugar | Ru5P (R5P) | 1871.72 | 8.60 | 1865.13 | 10.25 | 1863.59 | 8.76 |
| 61 | Phosphorylated sugar | F6P | 695.80 | 93.35 | 629.32 | 26.09 | 636.93 | 35.56 |
| 62 | Phosphorylated sugar | G6P | 335.08 | 119.87 | 284.49 | 101.27 | 256.10 | 41.35 |
| 63 | Phosphorylated sugar | S7P | 788.07 | 262.95 | 570.95 | 79.62 | 498.08 | 68.61 |
| 64 | Phosphorylated sugar | F1,6BP | 5201.27 | 1401.45 | 4188.83 | 752.47 | 3764.52 | 1058.80 |
| 65 | Phosphorylated compound | GA3P | 170.17 | 86.28 | 154.20 | 86.09 | 160.09 | 66.37 |
| 66 | Phosphorylated compound | *α*-glyceric phosphate | 1225.47 | 388.04 | 841.14 | 145.20 | 768.75 | 158.33 |
| 67 | Phosphorylated compound | 2-PGA (3-PGA) | 141466.60 | 19580.92 | 122705.57 | 11820.21 | 137117.04 | 25425.32 |
| 68 | Phosphorylated compound | Phosphoenolpyruvate | 409.94 | 28.02 | 405.55 | 19.64 | 380.25 | 14.33 |
| 69 | Vitamin | Pyridoxine | 50.06 | 5.26 | 45.23 | 5.98 | 34.77 | 7.05 |
| 70 | Vitamin | Riboflavin | 13.04 | 1.79 | 12.30 | 0.65 | 13.15 | 1.68 |
| 71 | Vitamin | Pantothenate | 2793.58 | 445.41 | 2495.93 | 719.67 | 1679.33 | 276.85 |
| 72 | Vitamin | Niacinamide | 24.41 | 5.78 | 22.58 | 9.73 | 17.81 | 7.27 |
| 73 | Vitamin | Choline | 713.09 | 166.98 | 382.08 | 120.61 | 238.09 | 69.25 |
| 74 | Vitamin | Thiamine | 168.91 | 45.75 | 145.51 | 34.19 | 170.72 | 57.23 |

**Table S7**. Metabolites significantly affected by TCDD exposure by 4 h treatment. The fold changes are given as the ratio of the average content in TCDD and in DMSO.

| Metabolites | Fold change (LC-MS $p$ < 0.05) | |
|---|---|---|
| | 1 nM | 10 nM |
| Aspartic acid | 1.09 | 1.43 |
| F1,6BP | 1.02 | 1.05 |
| G6P | 1.18 | 1.55 |
| Histidine | 1.13 | 2.19 |
| Isoleucine | 1.42 | 2.38 |
| Lactate | 1.82 | 2.36 |
| Malic acid | 1.3 | 2.03 |
| Methionine | 1.45 | 2.03 |
| Phenylalanine | 1.52 | 2.12 |
| Pyroglutamic acid | 0.94 | 1.32 |
| S7P | 1.25 | 1.19 |
| Threonine | 1.31 | 1.9 |
| Tyrosine | 1.46 | 2.48 |

**Table S8**. Metabolites significantly affected by TCDD exposure by 24 h treatment. The fold changes are given as the ratio of the average content in TCDD and in DMSO.

| **Metabolites** | **Fold change (LC-MS $p$ < 0.05)** | |
|---|---|---|
| | **1 nM** | **10 nM** |
| Acetyl CoA | 0.79 | 0.8 |
| Aconitic acid | 0.62 | 0.45 |
| Alanine | 0.59 | 0.44 |
| Arginine | 1.56 | 2.1 |
| Asparagine | 0.3 | 0.19 |
| Aspartic acid | 0.69 | 0.59 |
| ATP | 0.7 | 0.58 |
| cAMP | 0.78 | 0.77 |
| Choline | 0.54 | 0.33 |
| Citric acid | 0.92 | 0.89 |
| CMP | 0.77 | 0.83 |
| Creatinine | 0.79 | 0.71 |
| Cytidine | 0.84 | 0.8 |
| dGMP | 0.96 | 0.85 |
| FAD | 0.72 | 0.79 |
| GDP | 0.6 | 0.69 |
| Glutamine | 0.73 | 0.44 |
| Histidine | 0.71 | 0.43 |
| Hydroxyproline | 0.42 | 0.48 |
| Isoleucine | 0.65 | 0.54 |
| Lysine | 0.59 | 0.54 |
| Methionine | 0.62 | 0.52 |
| $NAD^+$ | 0.68 | 0.66 |
| NADH | 0.76 | 0.63 |
| Pantothenate | 0.89 | 0.6 |

| | | |
|---|---|---|
| Phenylalanine | 0.64 | 0.61 |
| Phosphoenolpyruvate | 0.99 | 0.93 |
| Proline | 0.62 | 0.68 |
| Pyroglutamic acid | 0.73 | 0.47 |
| S7P | 0.72 | 0.63 |
| Serine | 0.5 | 0.28 |
| Stearoyl-CoA | 0.74 | 0.55 |
| Threonine | 0.71 | 0.6 |
| Tryptophan | 0.67 | 0.59 |
| UMP | 0.7 | 0.63 |
| XMP | 0.62 | 0.72 |

**Figure S1**. **A)** Chromatography comparison for selected 12 metabolites on three LC gradients on a Waters BEH Amide column. **B)** Effect of changes in column temperature on selected polar metabolites. Waters BEH Amide column temperature was 20°C, 30°C, and 40°C. **C)** Comparison of chromatography for selected 12 metabolites using a Waters BEH Amide and Waters BEH HILIC columns (1.7 μm; 2.1 x 100 mm). **D)** Effect of increasing mobile phase buffer concentration on polar metabolites chromatogram. **E)** Effect of changes in pH value on selected polar metabolites. pH values were adjusted to 5, 7, and 9 by acetic acid or ammonium hydroxide. Mobile phase A consisted of 90:10 water:acetonitrile containing 20 mM ammonium acetate, Mobile phase B consisted of 10:90 water:acetonitrile containing 20 mM ammonium acetate. Unless otherwise stated, the pH of mobile phase A and B were adjusted to 9.0, Waters BEH Amide Column (1.7 μm; 2.1 x 100 mm) were employed and the temperature was set as 30°C.

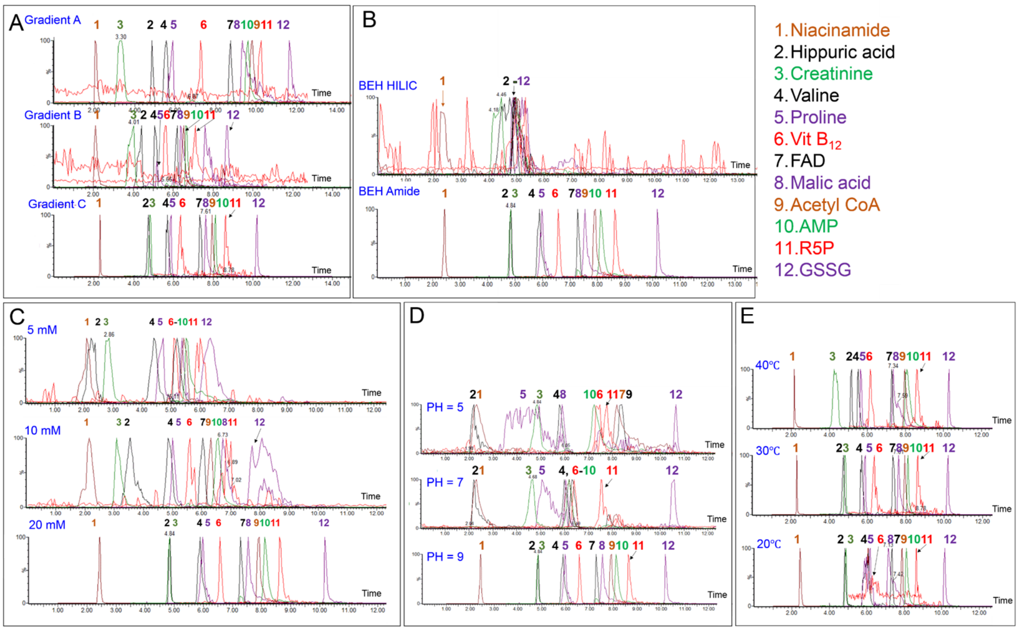

**Figure S2.** Separation of five pairs of isomers by gradient C. Processed using Waters BEH Amide column. Mobile phase A consisted of 90:10 water:acetonitrile containing 20 mM ammonium acetate (pH = 9.0). Mobile phase B consisted of 10:90 water: acetonitrile containing 20 mM ammonium acetate (pH = 9.0). Waters BEH Amide Column (1.7 μm; 2.1 x 100 mm) were employed and the temperature was set as 30°C.

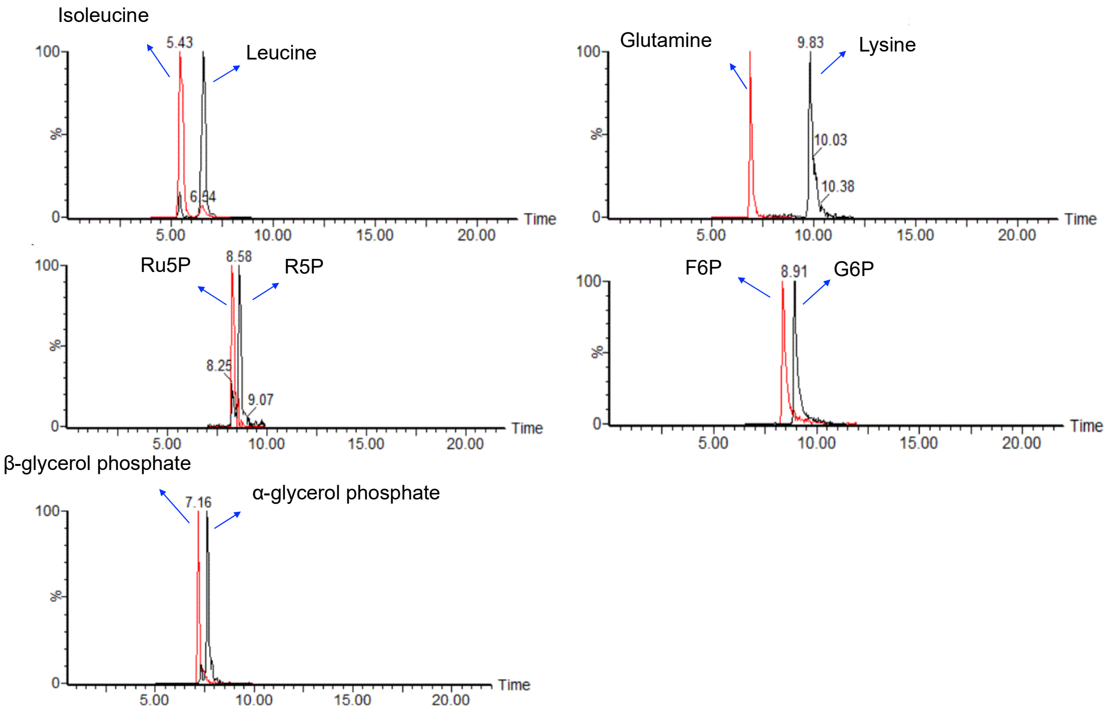

**Figure S3**. Pharmacologic targeting of aerobic glycolysis in Huh-7 cells. The glycolytic pathway (orange) and its metabolic interconnections with the pentose phosphate pathway (PPP) (blue) and the tricarboxylic acid (TCA) cycle (green) are shown. Pyruvate and glutamine were added in media to survive the cells. Red and blue color of the metabolites represent increasing or decreasing in treatment group comparing with control group, 3-BrPy blocked on HK-ii and GAPDH, most of the changes were in glycolytic pathway, indicating it was inhibited by 3-BrPy.

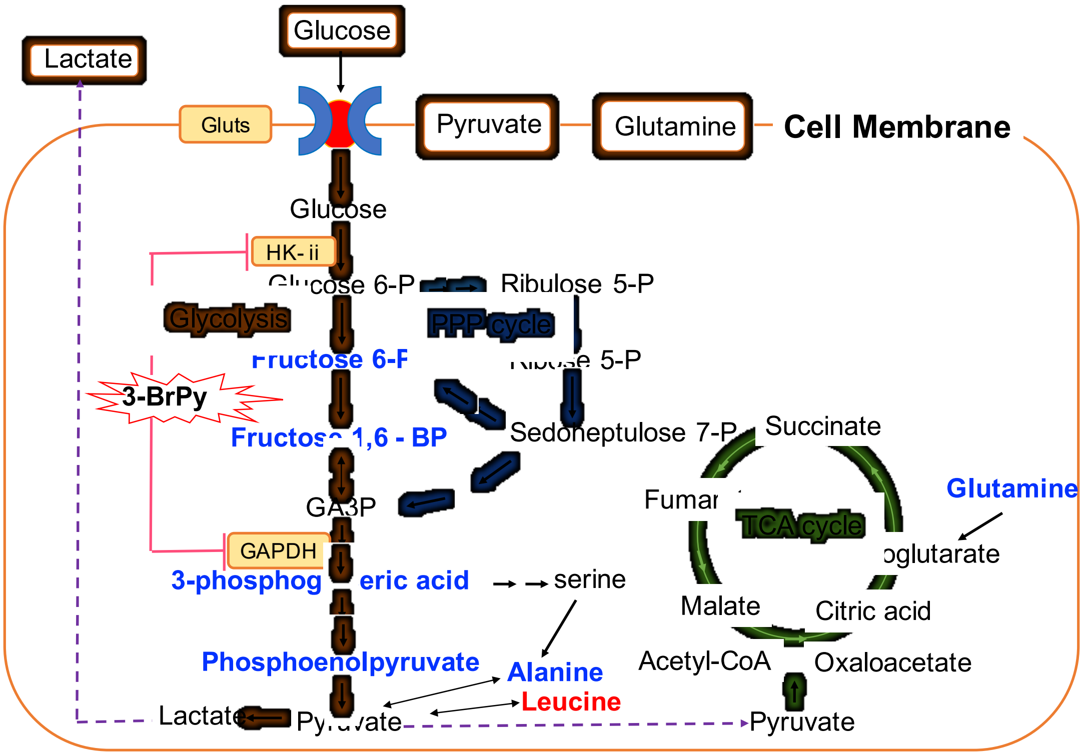

**Figure S4**. **A)** %Cytotoxicity of each group by MTT assay at 560 nm wave length. **B)** Scores plot of principal components analysis of polar metabolites in Huh-7 cells infected with different concentration of 3-BrPy. **C)** Variation patterns of polar metabolites involved in glycolysis metabolism in presence of 3-BrPy, representing the significant increasing (red) or decreasing (blue) quantitated metabolites. The same with Figure 2, The z-scores were imported into R and the heatmap.2 command from the G plots package was used to create the heatmaps. The metabolites were not sorted but were separated into groups based on metabolite category.

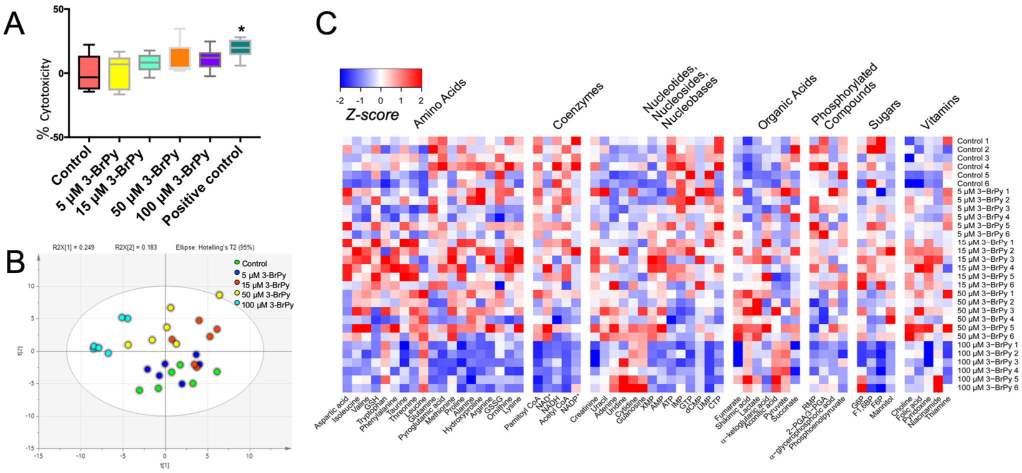

**Figure S5**. Concentration-dependent effects of 3-BrPy on metabolism of cultured Huh-7 cells. Metabolites are distributed in energy metabolism (NADH, ATP), oxidative stress (GSH, GSSG), glycolytic metabolism (F6P, F1,6BP, 3-phosphogluceric acid, phosphoenolpyruvate), TCA cycle (citric acid, succinate, malic acid), PPP cycle (S7P) and amino acid metabolism (glutamine, serine, alanine, leucine). Cells were incubated for 24 h with the concentration indicated. Controls: 0 µM 3-BrPy; positive controls: 2 mM glucose in media and 0 µM 3-BrPy. Significant difference among groups is analyzed by one-way ANOVA with Dunnett's test, *p*-value was adjusted for multiple comparisons. *, $p < 0.05$; **, $p < 0.01$; ***, $p < 0.001$; and ****, $p < 0.0001$. The cell number was normalized to 2.50E+06 per sample.

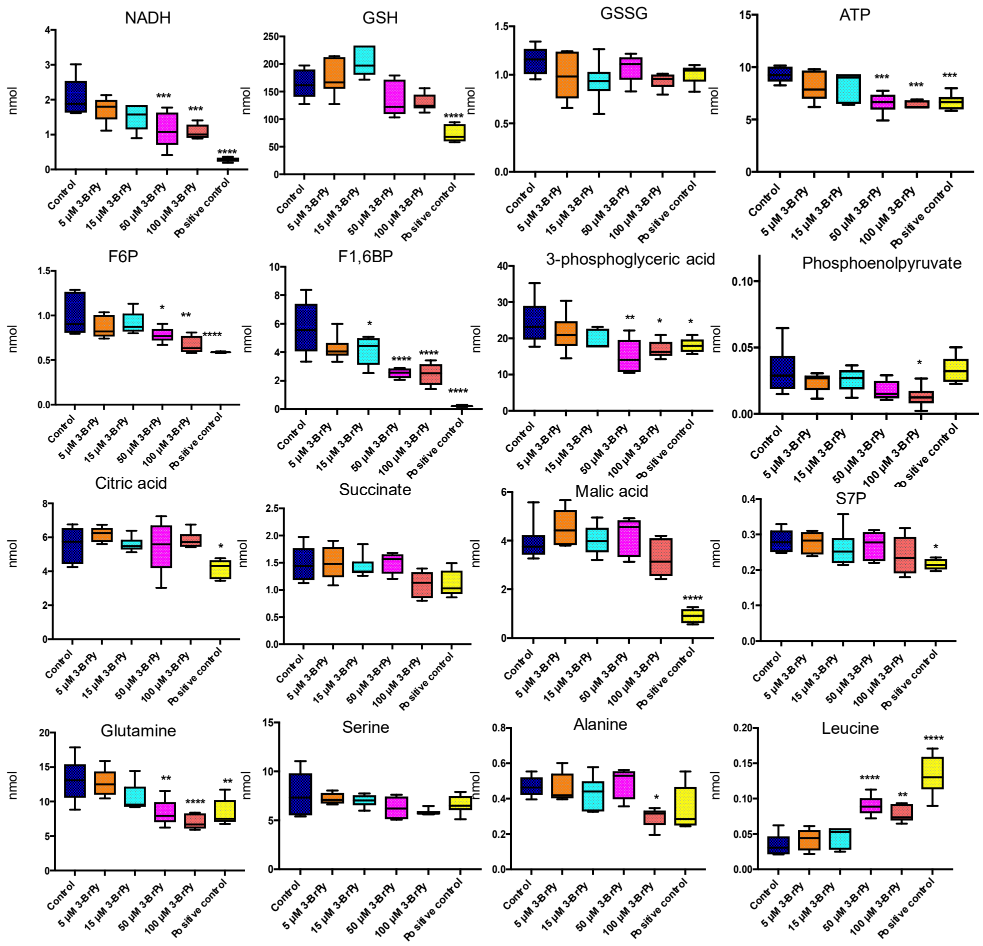

**Figure S6.** Protein concentration determination in Huh-7 Cells using the standard BCA Protein Assay.

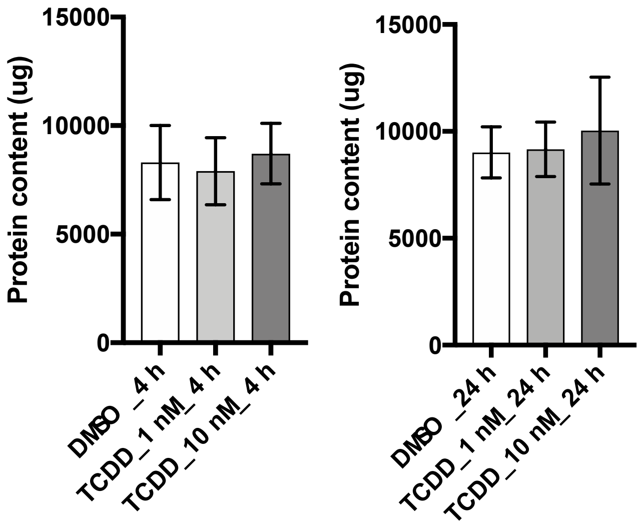

**Figure S7.** Scores plot of principal components analysis of polar metabolites in Huh-7 cells infected with 1 nM and 10 nM of TCDD in 4 h and 24 h treatment.

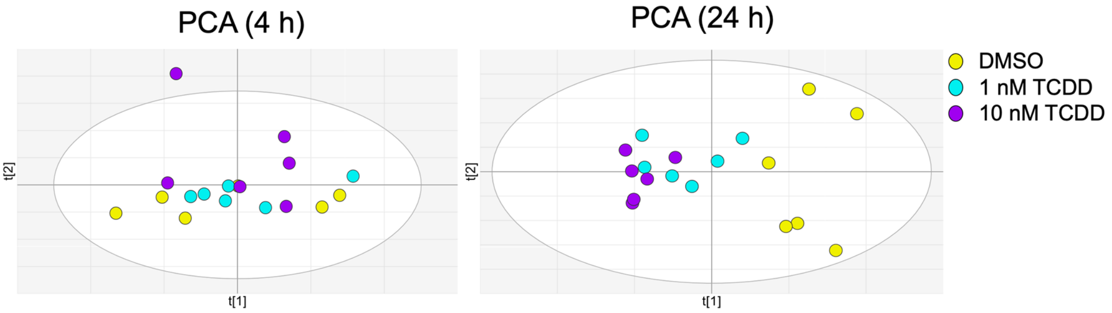

**Figure S8.** A) PCA plot of polar metabolites in Huh-7 cells treated with 1 and 10 nM TCDD for 24 h by $^1$H NMR. B) PLS-DA plot of polar metabolites in Huh-7 cells treated with 1 nM and 10 nM of TCDD for 24 h.

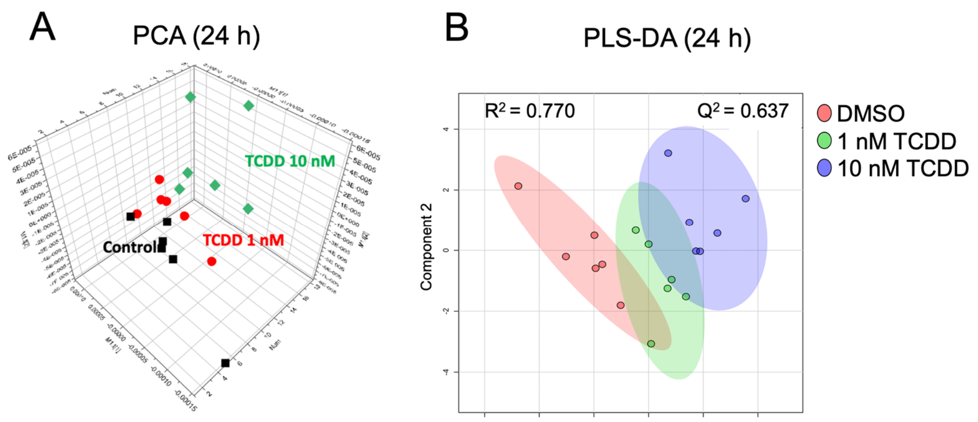

**Figure S9.** Concentration-dependent effects of TCDD on the metabolism of cultured Huh-7 cells as detected by $^{1}H$ NMR. Huh-7 cells were treated with 1 and 10 nM TCDD for 4 h and 24 h. DMSO (vehicle) served as control. Significant differences among groups was determined by one-way ANOVA with Dunnett's test, *p*-value was adjusted for multiple comparisons. *, $p < 0.05$; **, $p < 0.01$.

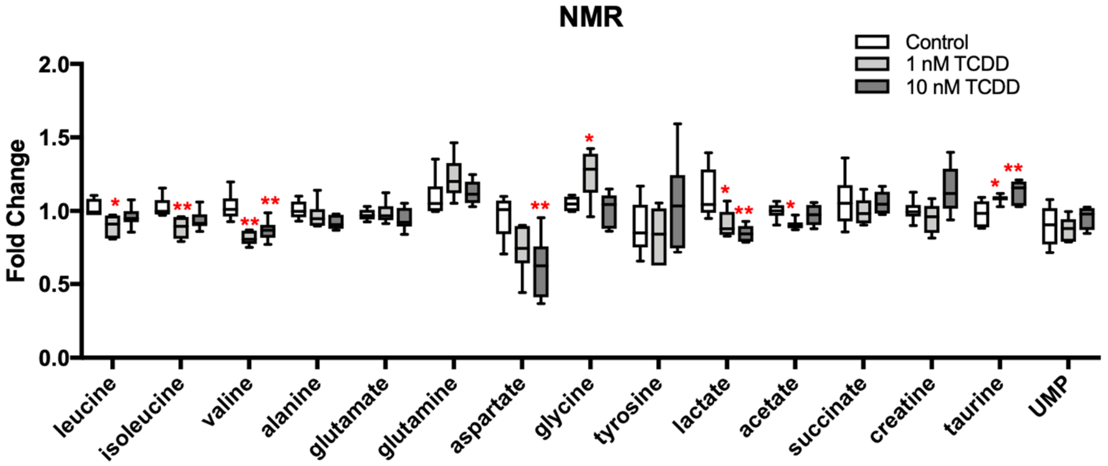

Supplement: Supplementary file 1 [file metabolites-09-00118-s001.pdf]
